# TP53/TAU axis regulates microtubule bundling to control alveolar stem cell–mediated regeneration

**DOI:** 10.1172/JCI194762

**Published:** 2026-02-05

**Authors:** Satoshi Konishi, Khaliun Enkhbayar, Shuyu Liu, Naoya Miyashita, Yoshihiko Kobayashi, Vera Hutchison, Ashna Sai, Pankaj Agarwal, Jonathan Witonsky, Nathan D. Jackson, Max A. Seibold, Jichao Chen, Aleksandra Tata, Purushothama Rao Tata

**Affiliations:** 1 Department of Cell Biology and; 2 Department of Surgery, Surgical Sciences, Duke University School of Medicine, Durham, North Carolina, USA.; 3 Department of Pulmonary Medicine, University of Texas MD Anderson Cancer Center, Houston, Texas, USA.; 4 Department of Pediatrics, University of California, San Francisco, San Francisco, California, USA.; 5 Center for Genes, Environment and Health and; 6 Department of Pediatrics, National Jewish Health, Denver, Colorado, USA.; 7 Division of Pulmonary Sciences and Critical Care Medicine, University of Colorado School of Medicine, Aurora, Colorado, USA.; 8 Department of Pediatrics, Perinatal Institute Division of Pulmonary Biology, University of Cincinnati and Cincinnati Children’s Hospital Medical Center, Cincinnati, Ohio, USA.; 9 Duke Regeneration Center, Duke University, Durham, North Carolina, USA.; 10 Division of Pulmonary, Allergy, and Critical Care Medicine, Department of Medicine, Duke University School of Medicine, Durham, North Carolina, USA.; 11 Center for Advanced Genomic Technologies, Duke University, Durham, North Carolina, USA.; 12 Duke Cancer Institute, Duke University School of Medicine, Durham, North Carolina, USA.

**Keywords:** Cell biology, Pulmonology, Cytoskeleton, Stem Cells, p53

## Abstract

Cells exhibit diverse sizes and shapes, tailored for functional needs of tissues. Lung alveoli are lined by large, extremely thin epithelial alveolar type 1 cells (AT1s). Their characteristic morphology is essential for lung function and must be restored after injury. The mechanisms underlying small, cuboidal alveolar type 2 cell (AT2) differentiation into thin AT1s remain elusive. Here, we demonstrated that AT2s undergo a stepwise morphological transformation characterized by the development of a unique thick microtubule (MT) bundle organization, critical for AT1 morphology. Using AT2 cultures and in vivo genetic loss-of-function models, we found that MT bundling occurred in a transitional cell state during AT2 differentiation and was regulated by the TP53/TAU (encoded by the microtubule-associated protein tau [*MAPT*] gene) signaling axis. Notably, TAU underwent a linear clustering process, forming beads-on-a-string-like pattern that preceded thick MT bundle formation. Genetic gain or loss of function of TAU in mouse or human models prevented the formation of thick MT bundles, highlighting the critical role of precise TAU levels in generating ultrathin AT1s. This defect was associated with increased tissue fibrosis following bleomycin-induced injury in vivo. GWAS analysis revealed risk variants in the MAPT locus in lung diseases. Moreover, TP53 controlled TAU expression and its loss phenocopied TAU deficiency. This work revealed an unexpected role for TAU in organizing MT bundles during AT2 differentiation.

## Abstract 2

This work uncoreved a unique thick microtubule (MT) bundle organization which is required to generate expansive and thin cell morphology of during AT2s differentiation into AT1s.

## Graphical abstract

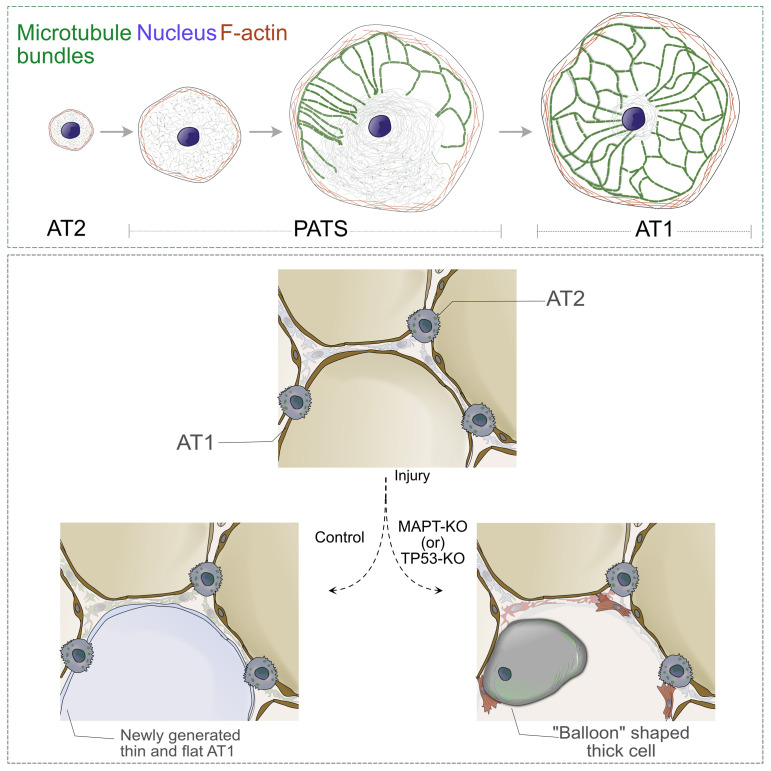

## Introduction

Tissues must maintain proper cellular composition and morphological organization to carry out their functions. Defects in either cellular composition or structure have been implicated in various diseases, such as cancers, organ fibrosis, and tissue atrophy (1–4). Therefore, it is essential to understand the mechanisms that allow cells to achieve their appropriate identity and often complex cell morphology during development, homeostasis, and repair. In the lung, the gas-exchanging alveoli have an extremely thin epithelial lining that both facilitates diffusion of gases and serves as a barrier (5). About 95% of this lining is occupied by alveolar type 1 cells (AT1s), one of the thinnest cell types in the human body (6–9). The remaining area is occupied by the apical domains of small cuboidal alveolar type 2 cells (AT2s), which serve as facultative stem cells that can self-renew and differentiate into AT1s both at homeostasis and after injury.

Multiple growth factor signaling and transcriptional regulators have been implicated in AT2-to-AT1 differentiation during development and regeneration (10–25). Additionally, cells must coordinate structural components, such as actin, microtubules (MTs), and cytokeratin, to provide a cytoskeleton to build and support the cell body. Indeed, recent studies have implicated actin-dependent biophysical forces mediated by breathing movements and CDC42 mechanical stretch in the maintenance of AT1 identity or AT2-to-AT1 differentiation, respectively (18, 26). Additionally, integrins and cytokeratins have been shown to play critical roles in AT2-to-AT1 differentiation via regulation of immune cell–mediated alveolar epithelial repair processes (27, 28). Previous studies have demonstrated that AT2-to-AT1 differentiation involves a transitional state (also known as pre-alveolar type 1 transitional states [PATS], DATPs, or KRT8^hi^ ADIs), of which the abnormal induction or persistence can induce fibrotic responses in alveolar fibroblasts, leading to pulmonary fibrosis (29–33). Nevertheless, the relationship between programs that drive PATS and those that effect morphological changes remain elusive. Specifically, little is known about the transcriptional programs that guide structural components to shape the thin, expansive morphology of AT1s.

Here, we show that AT2-to-AT1 differentiation is associated with an MT bundling process that is essential for them to acquire large and thin morphology. Specifically, using a newly optimized 2-dimensional (2D) culture model, we have uncovered a dynamic process in which individual radial MTs are remodeled to generate thick MT bundles. This process is mediated by TAU (encoded by the microtubule-associated protein tau [*MAPT*] gene), which is highly expressed in PATS and AT1s and localized to thick MT bundles. Genetic gain or loss of function of TAU leads to disorganization of MTs, loss of thick MT bundles, and disruption in AT1 generation both ex vivo and in vivo. Furthermore, we show that loss of function of the transcription factor TP53 regulates TAU and recapitulates phenotypes observed in TAU-mutant cells.

## Results

### Newly optimized conditions for AT2 maintenance and differentiation in 2D cultures.

**Figure 1:**
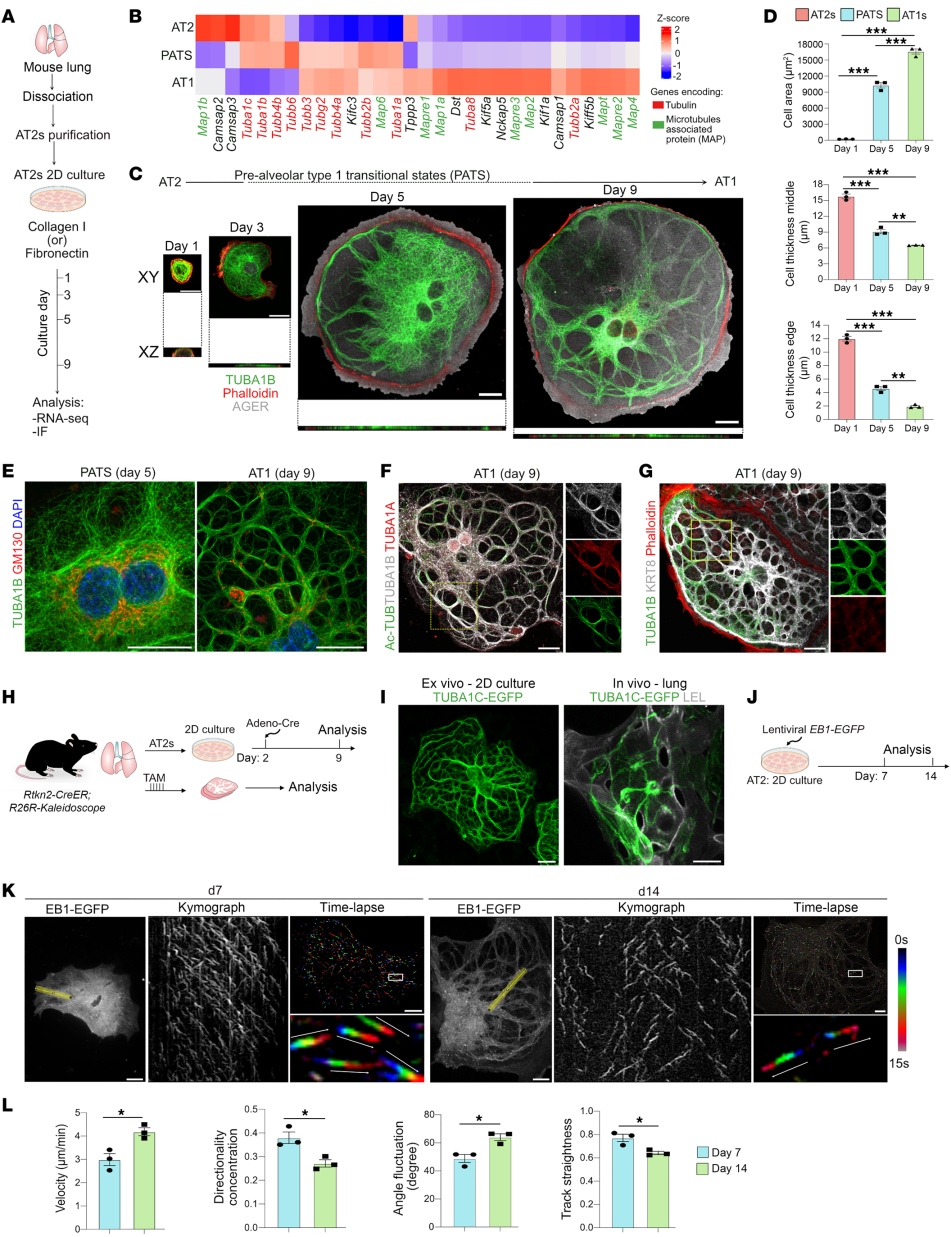
Alveolar stem cell MTs undergo dynamic changes to form thick MT bundles during differentiation. (**A**) Experimental design for mouse AT2 isolation, culture, and sample collection. IF, immunofluorescence. (**B**) Heatmap shows expression of tubulin-encoding and MAP-encoding genes in cultured AT2s, PATS, and AT1s. (**C**) Staining for TUBA1B (green), phalloidin (red), and AGER (gray) on cells cultured on fibronectin showing AT2-PATS-AT1 cell fate and cell morphology transition. Scale bar: 20 μm. (**D**) Quantification of area and thickness (in the middle and edge) of alveolar epithelial cells on days 1, 5, and 9 of culture. **P < 0.005, ***P < 0.001, 1-way ANOVA. *n* = 3 biological replicates. (**E**) Staining for TUBA1B (green) and GM130 (red) at indicated times. DAPI stains nuclei (blue). Scale bar: 20 μm. (**F**) Staining for tubulin proteins in AT2s cultured on fibronectin for 9 days. Scale bar: 20 μm. Ac-TUB, acetylated tubulin. (**G**) Staining for TUBA1B (green), KRT8 (gray), and phalloidin (red) in AT1s. Scale bar: 20 μm. (**H**) Experimental design for ex vivo and in vivo AT1-specific tubulin lineage tracing in *Rtkn2-CreER R26R-Kaleidoscope* mice. (**I**) Images showing TUBA1B-EGFP (green) and LEL (*Lycopersicon Esculentum Lectin*, gray) in cultured AT1s and in vivo lungs. Scale bars: 20 μm. (**J**) Experimental workflow for AT2 infection with *EB1-EGFP* lentivirus followed by live imaging on day 7 and day 14. (**K**) Kymograph and time-lapse images illustrating tubulin dynamics and orientation in cells on day 7 and day 14. White box indicates region of enlarged image. White arrows indicate direction of growing plus ends of MTs. Scale bars: 20 μm. (**L**) Quantification of EB1-EGFP comet velocity (μm/min), directionality concentration, angle fluctuation (degree), and track straightness in cells cultured for 7 and 14 days. *P < 0.05, unpaired 2-tailed *t* test. Data in **D** and **L** are presented as mean ± SEM. *n* = 3 biological replicates.

To assess morphological dynamics during mouse AT2-to-AT1 differentiation, we sought to optimize 2D cultures that enable efficient cell state transitions. Previous studies have demonstrated that AT2s can be cultured in 50% Matrigel (10, 34). To establish a 2D culture model, AT2s were first plated on 5% Matrigel–coated wells. However, even at later times, both large and thin cells that expressed AGER (AT1 marker) and ABCA3 (AT2 marker) were observed, suggesting incomplete differentiation (Supplemental Figure 1A; supplemental material available online with this article; https://doi.org/10.1172/JCI194762DS1). On Collagen I–coated plates, tightly packed colonies of cells expressing ABCA3 and SFTPC were present throughout the culture duration (Supplemental Figure 1B). By contrast, culture on fibronectin-coated plates revealed the presence of CLDN4^+^ PATS and large and thin AGER^+^ AT1-like cells at early (day 5) and later (day 9) times (Figure 1A and Supplemental Figure 1C). To visualize the morphological dynamics during AT2-to-AT1 differentiation, we performed time-lapse live imaging of cells cultured on fibronectin starting day 3 for 72 hours. Our data revealed gradual stretching of AT1-like cells with the appearance of arborizing cytoskeletal components from day 6 that were maintained throughout the culture duration (Supplemental Figure 1D and Supplemental Video 1). We then performed bulk RNA-seq on cells collected from Collagen I– (AT2s) and fibronectin-coated plates harvested on day 5 (PATS) and day 9 (AT1s) (Figure 1A). As expected, differential gene expression analysis revealed previously reported AT2 (*Sftpa1*, *Abca3*, *Sftpc*, *Lamp3*), PATS (*Krt8*, *Sfn*, *Sox4*), and AT1 (*Hopx*, *Aqp5*, *Ager*, *Cav1*) markers in different culture conditions (Supplemental Figure 1E). Together, we established a 2D culture system to maintain AT2s and their differentiation to AT1s in defined conditions.

### Transcriptome profiling reveals dynamic expression pattern of structural and regulatory components of MTs during AT2-to-AT1 differentiation.

Our above live imaging data revealed the appearance of arborizing cytoskeletal structures. To further evaluate these structures, we analyzed the above transcriptome data and found expression of transcripts related to both structural (*Tuba1c*, *Tuba1b*, *Tuba1a*) and regulatory (*Map6*, *Mapre3*, *Map1a*, *Map2*, *Kif1a*, *Camsap1*) components of MT assembly in specific cell types. We found enrichment of multiple structural and regulatory components of MTs in PATS and AT1s indicating that MTs undergo significant reorganization during AT2 differentiation to AT1 via PATS (Figure 1B). To assess whether such changes occur during AT2 differentiation in vivo, we reevaluated previously generated single-cell RNA-seq (scRNA-seq) data from bleomycin-induced lung injury (30). We found enrichment of *Tubb2b*, *Tubb5*, *Tubb6*, *Map1b*, *Map4*, and *Map7* in PATS, whereas *Tuba1a*, *Tuba8*, *Tubb2a*, *Tubb4b*, *Map2*, and *Map6* were enriched in AT1s (Supplemental Figure 2A). Together, transcriptome data revealed dynamic expression of MT components during AT2-to-AT1 differentiation both in vivo and ex vivo.

### MTs undergo dynamic reorganization and generate thick bundles during AT2-to-AT1 differentiation ex vivo and in vivo.

We next performed immunostaining to visualize expression and localization of MTs, actin, and cytokeratins during AT2-to-AT1 differentiation. AT2s had a dense network of individual radial MT fibers distributed throughout the cell body. By contrast, AT1s had organized, thick, bundle-like structures, each composed of multiple individual MT fibers (Figure 1C and Supplemental Figure 2B). Interestingly, MT changes correlated with an increase in cell area and decrease in cell thickness (Figure 1D). Immunostaining for TUBA1B revealed a dynamic change in its localization as AT2s differentiated to AT1s via PATS. On day 5, we observed the emergence of individual thick MT bundles, whereas on day 9, cells had a highly branched network of thick bundles all around the cell body as the cells matured to AT1s (Figure 1C). In most cell types, MTs are anchored to the perinuclear Golgi via MT organizing centers (35, 36). To assess Golgi localization, we performed immunostaining for GM130 (37). The Golgi apparatus was restricted to perinuclear regions in PATS, whereas it was co-localized with thick MT bundles including at branch points in AT1s, suggesting that the Golgi serves as an anchoring point for MTs, as in neuronal axons (38, 39) (Figure 1E). Additionally, staining for PK-mito, LAMP1, and CANX, which marks mitochondria, lysosomes, and ER, respectively, revealed that these organelles co-localized with thick MT bundles (Supplemental Figure 2C). Furthermore, immunostaining for acetylated tubulin (Ac-TUB), a marker of the stabilized form of MTs, revealed that thick MT bundles correlated with mature forms of MTs (40) (Figure 1F). Of note, immunostaining for TUBA1A, TUBA1B, and MAP2 showed thick MT bundles composed of multiple tubulin classes and MAPs (Figure 1F and Supplemental Figure 2D). Among the actin and intermediate filaments, KRT8 localization overlapped with tubulins within the thick MT bundles whereas actin (phalloidin) was highly enriched in the cortex and the basal side of the cells (Figure 1, C and G, and Supplemental Figure 2E).

To assess whether the thick MT bundles observed in 2D cultures are also present in AT1s in vivo, we utilized the *Rtkn2-CreER R26R-Kaleidoscope* (hereafter referred to as *Rtkn2-Kaleidoscope*) mouse line, which expresses TUBA1C fused to green fluorescent protein (EGFP) (41). Administration of tamoxifen (in vivo) or Adeno-Cre virus (ex vivo) activates the expression of TUBA1C-EGFP, thereby enabling the localization of tubulins specifically in AT1s (Figure 1H). To assess TUBA1C-EGFP protein localization in cultured cells, we purified AT2s from *Rtkn2-Kaleidoscope* mice and cultured them as described above (Figure 1H). As expected, we found radial distribution of TUBA1C-EGFP throughout the cell body in AT2s from *Rtkn2-Kaleidoscope* mice whereas AT1s exhibited EGFP localization in a pattern similar to that of thick MT bundles (Figure 1I and Supplemental Figure 2F). To assess the tubulin localization pattern in vivo, lungs were collected from tamoxifen-administered *Rtkn2-Kaleidoscope* mice followed by thick tissue sectioning and imaging to visualize large, flat, and thin AT1s in alveolar sacs. Confocal imaging followed by maximum-intensity projection revealed EGFP localization consistent with AT1s having thick MT bundles in vivo (Figure 1I).

MTs are polar structures with a fast growing plus end and a slowly growing minus end that collectively provide the directionality of MT growth (42). We utilized end-binding 1–EGFP (EB1-EGFP), which allows tracking of MT plus ends, to assess MT growth directionality and kinetics in real time. 2D-cultured mouse AT2s were transduced with lentiviral *EB1-EGFP* followed by live imaging at early (day 7) and late stages (day 14) of differentiation to capture these dynamics in PATS and AT1s, respectively (Figure 1J and Supplemental Videos 2 and 3). Time-lapse imaging and comet tracking revealed that cells at day 7 showed unidirectional movement from center to cortex, whereas cells from day 14 showed bidirectional growth. This finding was confirmed by kymograph-based quantification analysis (Figure 1K and Supplemental Videos 2 and 3). These data suggest that alveolar epithelial cells shift their MT growth from unidirectional to bidirectional as the AT2s differentiate into large and thin AT1s. Moreover, an increase in EB1 comet velocity and angle fluctuation on day 14 indicated enhanced MT dynamics and polymerization and switching directions within bundled tracks (Figure 1L). Additionally, a decrease in directionality concentration, and track straightness at day 14 compared with day 7, suggested the emergence of bidirectional movement along bundled MTs during PATS-to-AT1 transition (Figure 1L). Together, AT2-PATS-AT1 differentiation processes can be recapitulated in our 2D ex vivo culture system, revealing a unique thick MT bundle organization pattern in AT1s.

### Dynamic expression and localization of TAU during AT2 differentiation.

**Figure 2:**
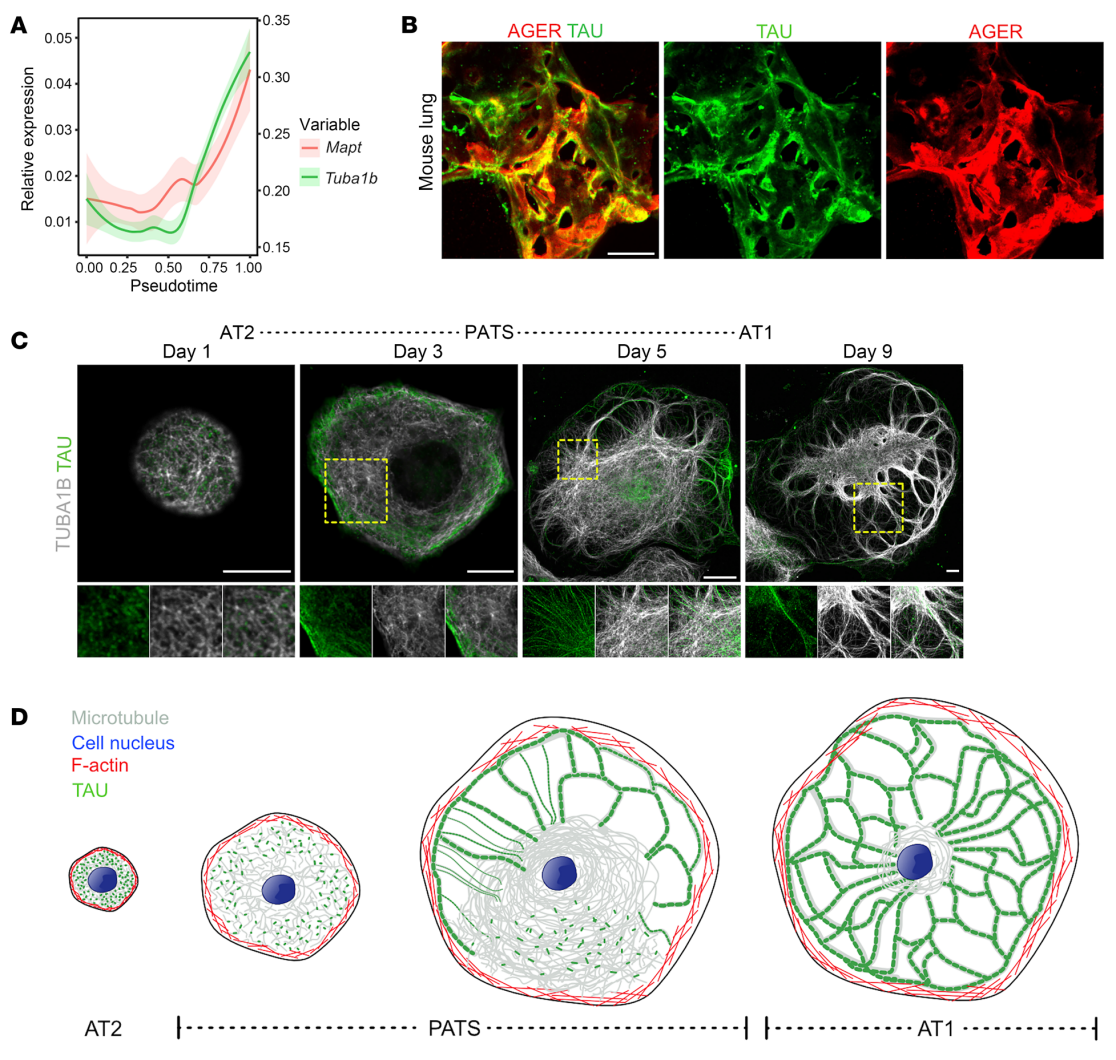
Dynamic expression and localization of TAU precede thick MT bundle formation during AT2 differentiation. (**A**) Pseudotime analysis visualizing gene expression dynamics of *Mapt* and *Tuba1b* during AT2-to-AT1 differentiation. (**B**) Immunostaining for AGER (red) and TAU (green) in the alveolar region of a thick tissue section showing TAU localization in AT1s. Scale bar: 20 μm. (**C**) Staining for TUBA1B (gray) and TAU (green) at indicated times of culture. Scale bars: 20 μm. Yellow box indicates region of single-channel images. (**D**) Schematic showing the expression and organization of TAU, MTs, and F-actin during AT2-to-AT1 differentiation.

The above data revealed that MT components and associated genes are differentially expressed during AT2-to-AT1 differentiation. Among these, MAPs are known to directly bind MTs and facilitate their nucleation in neurons and oligodendrocytes (43). To evaluate the expression of MAPs, we plotted relative expression of relevant genes in a pseudotime trajectory encompassing AT2s, PATS, and AT1s using time series scRNA-seq data that captured cellular dynamics at different times following bleomycin-induced lung injury (30). Unexpectedly, we found that *Mapt* (encoding TAU), a gene that has been extensively studied in Parkinson’s and Alzheimer’s diseases, is dynamically expressed during AT2-to-AT1 differentiation (44, 45). Specifically, *Mapt* expression gradually increased as AT2s transitioned to PATS, with the highest expression in AT1s (Figure 2A). Furthermore, this expression pattern correlated with that of MT components, including *Tuba1b*, suggesting that TAU plays a role in assembling MTs. To validate its expression in AT1s in vivo, we performed co-immunostaining for TAU and AGER on thick tissue sections followed by imaging and maximum-intensity projection (Figure 2B). To further evaluate its expression and localization dynamics, we carried out co-immunostaining for TAU and TUBA1B on cells collected at different times during AT2-to-AT1 differentiation. In line with transcriptome data, we found a gradual increase in TAU levels as AT2s differentiate into AT1s via PATS (Figure 2C and Supplemental Figure 3A). Although it is expressed at low levels in AT2s, TAU shows a punctate localization pattern throughout the cell body. Notably, the localization changed to an organized fiber-like pattern as AT2s transitioned to PATS. Super-resolution imaging revealed that multiple TAU puncta are organized into a beads-on-a-string-like pattern in PATS and in mature AT1s (Supplemental Figure 3B). Interestingly, the fiber-like pattern resembled the thick MT bundle pattern even in the absence of clear bundles of TUBA1B, suggesting that TAU fibers precede MT bundle formation. At later times, dense, thick MT bundles are formed in mature AT1s. These data suggest a model in which TAU is organized into a string-like pattern that precedes thick MT bundle formation during AT2-to-AT1 differentiation (Figure 2D).

### Loss or gain of TAU disrupts thick MT bundle formation and AT1 thickness ex vivo.

**Figure 3:**
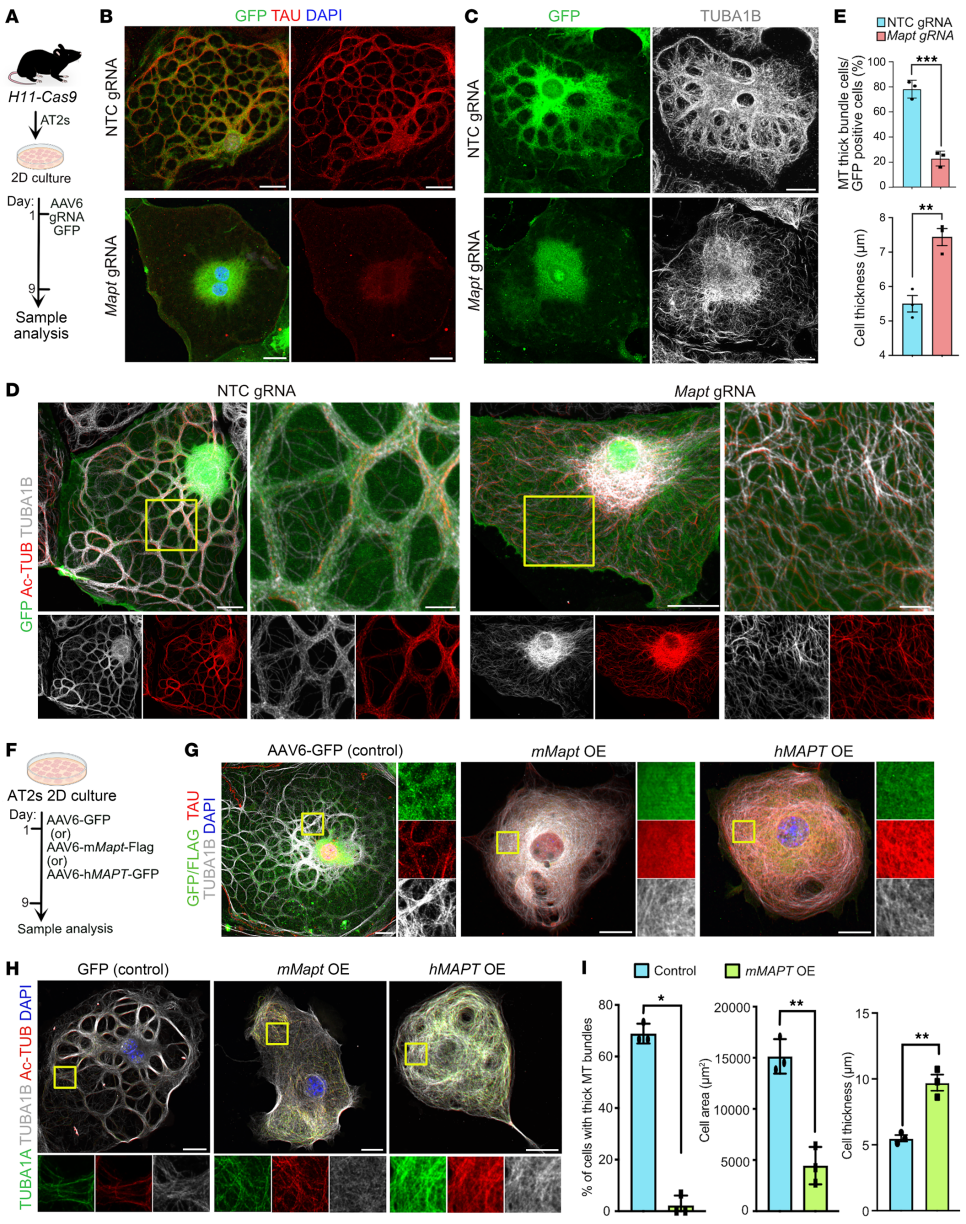
TAU regulates the formation of thick MT bundles. (**A**) Experimental design for AT2 isolation from *H11-Cas9* mice followed by AT2 culture and *AAV6-gRNA-GFP* infection to knock out *Mapt* ex vivo. (**B**) Staining for GFP (green, infected cells) and TAU (red) in NTC and *Mapt-KO* cells. Scale bars: 20 μm. (**C**) Staining for GFP (green) and TUBA1B (gray) in control and *Mapt*-KO cells. Scale bars: 20 μm. (**D**) Staining for TUBA1B (gray) and Ac-TUB (red) in infected GFP^+^ (green) control and *Mapt*-deleted cells. Scale bars: 20 μm (low magnification); 5 μm (high magnification). Yellow box indicates region of single-channel images. (**E**) Quantification of cells exhibiting thick MT bundles and cell thickness in control and *Mapt*-deleted cells. **P = 0.0049, ***P = 0.0005, unpaired 2-tailed *t* test. *n* = 3 biological replicates. (**F**) Schematic of ex vivo–cultured AT2s infected with mouse *Mapt* or human *MAPT* and analyses at indicated time point. (**G**) Staining for TAU (red) and TUBA1B (gray) in control and TAU-overexpressed (-OE) cells (green). Scale bars: 20 μm. Yellow box indicates region of single-channel images. DAPI stains nuclei (blue). (**H**) Staining for TUBA1A (green), TUBA1B (gray), and Ac-TUB (red) in TAU-OE and control cells. Yellow box indicates region of single-channel images. Scale bars: 20 μm. (**I**) Quantification of cell area, cell thickness, and the percentage of infected cells exhibiting thick MT bundles. *P = 0.05, Mann-Whitney *U* statistical test. **P < 0.005, unpaired 2-tailed *t* test. *n* = 3 biological replicates. Data in **E** and **I** are presented as mean ± SEM.

To assess the role of TAU during AT2 differentiation, we performed CRISPR-based *Mapt* knockout in purified AT2s in culture. First, we screened for efficient gRNAs selected from a previously described mouse Brie genome-wide gRNA library (46). Of the 4 gRNAs screened, 2 gave knockout efficiencies of 97% (gRNA1) and 67% (gRNA4) as assessed by ICE analysis (47) (Supplemental Figure 4, A and B). Then we generated adeno-associated viral 2/6 (AAV6) particles expressing gRNAs and GFP followed by transduction into AT2s purified from *H11-Cas9* mice and harvested cells for analysis on day 9 postinfection (48). AAV6 co-expressing nontargeting control (NTC) gRNA and GFP served as a control. Co-immunostaining for TAU and GFP (infected cells) revealed efficient deletion of the gene in *Mapt* gRNA–infected cells but not in controls (Figure 3, A and B, and Supplemental Figure 4, C and D). As expected, *Mapt* gRNA–infected GFP^+^ cells lacked thick MT bundles compared with NTC gRNA–infected cells. Immunostaining for GFP and TUBA1B and Ac-TUB revealed disorganized MTs dispersed throughout the cell body in *Mapt* gRNA1–infected cells, further validating the above observations (Figure 3, C and D). Quantification revealed a significant decrease in the number of cells with thick MT bundles in *Mapt* gRNA1 cells compared with NTC gRNA. Furthermore, we found a significant increase in the apical-basal thickness of *Mapt* gRNA1– versus NTC gRNA–infected cells (Figure 3E). We observed similar phenotypes using *Mapt* gRNA4 (Supplemental Figure 4E).

Previous studies using in vitro reconstitution assays revealed that a fine balance in the levels of TAU is essential for its proper assembly, localization, and MT organization (49, 50). To assess whether an increase in TAU levels affects MT bundle formation during AT2-to-AT1 differentiation, we ectopically expressed TAU in AT2s. Full-length *Mapt* coding sequence from mouse fused with FLAG-tag was used to generate AAV6-mouse *Mapt-*Flag vectors. Similarly, human full-length *MAPT* was cloned into a plasmid expressing GFP and was used to generate AAV6-human *MAPT-*GFP virus. AAV6-GFP served as a control (Figure 3F). Co-immunostaining for GFP/FLAG, TAU, TUBA1B, Ac-TUB, and TUBA1A revealed disorganized MTs in both mouse and human TAU gain-of-function conditions compared with controls (Figure 3, G and H, and Supplemental Figure 5A). Quantification further revealed a significant loss of thick MT bundles in MAPT gain-of-function cells compared with controls. Additionally, *Mapt* gain-of-function cells showed a significant decrease in cell area and increase in cell thickness, a phenotype similar to that seen in *Mapt* loss of function (Figure 3, A–I). In certain brain tauopathies, a mutation in TAU at amino acid position 301 with proline-to-lysine substitution is known to have gain-of-function activity and to disrupt MT organization (51–53). Therefore, we ectopically expressed a pathological form of TAU (TAU^P301L^) co-expressing GFP in mouse AT2s during their differentiation. Immunostaining for TUBA1B, TAU, GFP, TUBA1A, and Ac-TUB revealed disorganization of tubulins and lack of thick MT bundles in GFP^+^ cells (Supplemental Figure 5B). Further, to assess whether MT bundles are essential for maintaining AT1 cell thickness, we deleted or ectopically expressed *Mapt* once MT bundles were established in cultured AT1s. To do so, we first generated AT1s followed by delivery of *Mapt* gRNA or *mMapt*-OE on day 9, at which point the AT1s established MT bundles. Immunostaining for Ac-TUB and quantification of MT bundles on day 6 after gRNA delivery revealed that *Mapt* gRNA– and *Mapt*-OE–transduced cells lacked MT bundles (Supplemental Figure 5, C and D). Strikingly, we found a significant increase in cell thickness in *Mapt* gRNA and *Mapt*-OE cells compared with controls (Supplemental Figure 5E). Collectively, these data suggest that both loss and gain of TAU function alter MT bundle formation and AT1 cellular organization.

### TAU is required for proper organization of cells during AT2-to-AT1 differentiation in vivo.

Next, we sought to study the role of TAU in vivo utilizing a previously described constitutive *Mapt* deletion (*Mapt-KO*) mouse model (54) and assessing AT2-to-AT1 differentiation after bleomycin-induced lung injury. To assess the morphology of cells derived from AT2s, we specifically labeled AT2s with GFP using AAV5-GFP virus in control and *Mapt-KO* mice prior to bleomycin administration (55). This approach also allowed us to identify regions undergoing repair in response to bleomycin-induced injury (Supplemental Figure 6A). Co-immunostaining for GFP and AGER on thick tissue sections revealed large, flat, and thin AGER^+^ AT1s derived from GFP^+^ AT2s in control lungs. As expected, the confocal single stack showed that GFP-labeled AT1s in control lungs exhibited a thin cell morphology. In contrast, *Mapt-KO* lungs showed thick and balloon-shaped GFP^+^ cells that extruded into the alveolar lumina and lacked AT1 markers (Supplemental Figure 6B). Further assessment revealed a significant decrease in the number of thin cells (0–6 μm) and an increase in thick cells (13–40 μm) in *Mapt-KO* compared with controls (Supplemental Figure 6C).

**Figure 4:**
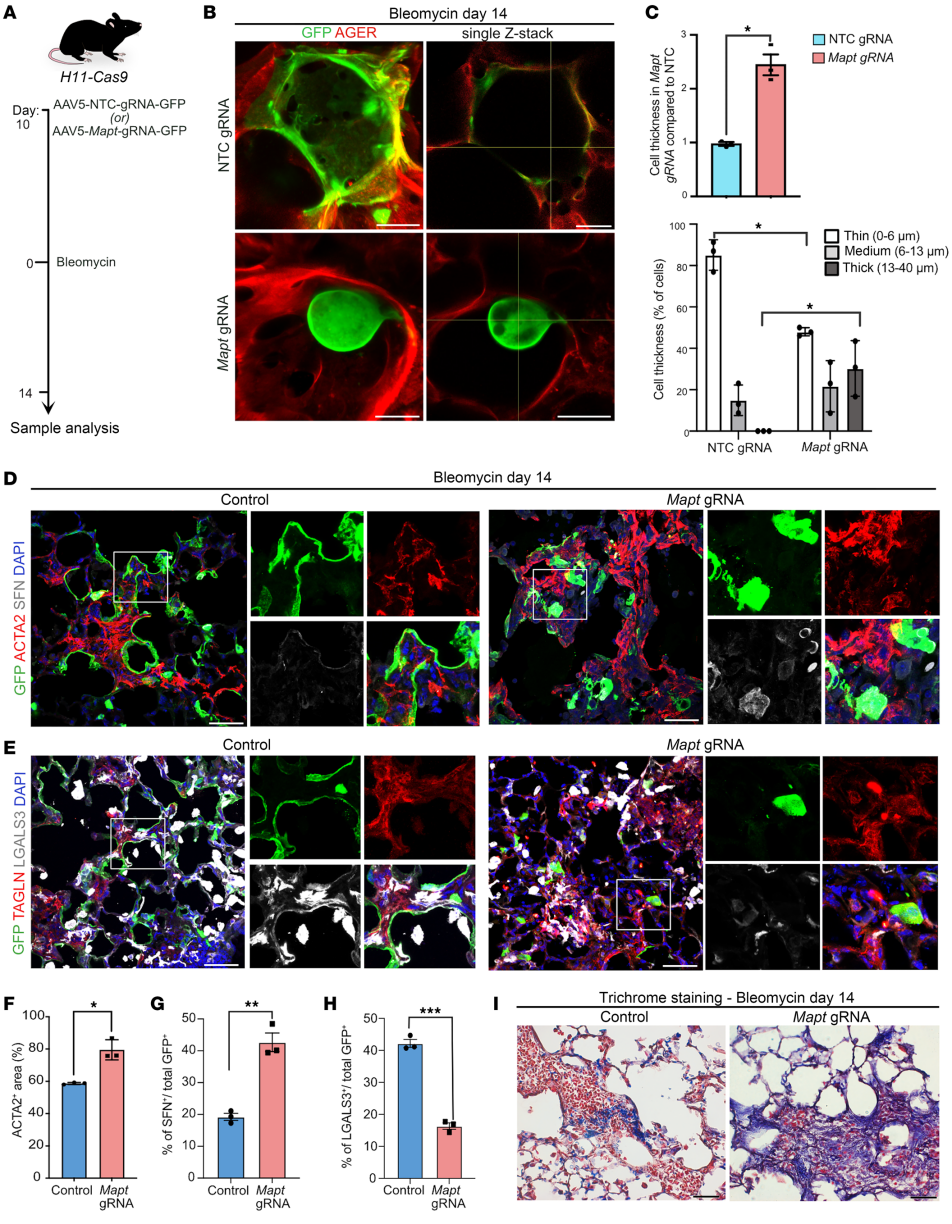
Loss of *Mapt* leads to abnormal cell organization and increased fibrosis in response to bleomycin injury. (**A**) Schematic of AT2-specific gRNA delivery to *H11-Cas9* mice followed by bleomycin injury and sample collection. (**B**) Staining for GFP (green, gRNA-delivered AT2s) and AGER (red) in control and *Mapt*-deleted cells after bleomycin injury. Scale bars: 20 μm. (**C**) Quantification of cell thickness and the distribution of GFP^+^ cells with different thickness in control and AT2-specific *Mapt-KO* lungs after bleomycin injury. *P < 0.05, unpaired 2-tailed *t* test. (**D**) Staining for GFP (green), ACTA2 (red), and SFN (gray) in controls and *Mapt*-deleted AT2s after bleomycin injury. Scale bars: 50 μm. DAPI stains nuclei (blue). (**E**) Staining for GFP (green), TAGLN (red), and LGALS3 (gray) in controls and *Mapt*-deleted AT2s after bleomycin injury. Scale bars: 50 μm. (**F**) Quantification of ACTA2^+^ area in bleomycin-injured lungs. *P < 0.05, unpaired 2-tailed *t* test. (**G**) Quantification of SFN^+^ cells among GFP^+^ cells in bleomycin-injured lungs. **P < 0.005, unpaired 2-tailed *t* test. (**H**) Quantification of LGALS3^+^ cells among GFP^+^ cells in bleomycin-injured lungs. ***P < 0.001, unpaired 2-tailed *t* test. (**I**) Trichrome staining on lungs collected from bleomycin-injured controls and *Mapt-*deleted mice. Scale bars: 100 μm. Data in **C** and **F**–**H** are presented as mean ± SEM. *n* = 3 biological replicates.

To exclude the possibility of non–cell-autonomous effects in the above experiments, we induced CRISPR-based loss of *Mapt* function specifically in AT2s. For this, we generated AAV5 virus carrying *Mapt* or NTC gRNAs and a green fluorescent protein (GFP marks infected cells) and administered them intranasally into *H11-Cas9* mouse lungs prior to bleomycin-induced injury (Figure 4A). As expected, co-immunostaining for GFP and AGER followed by imaging of thick tissue sections revealed large, thin, and flat cells co-expressing these markers in NTC gRNA lungs. However, *Mapt* gRNA–transduced cells showed a thick and balloon-shaped morphology and protruded into alveolar lumina (Figure 4B). Quantification further revealed a significant decrease in the number of thin cells (0–6 μm) and an increase in thick cells (13–40 μm) in *Mapt* gRNA–administered lungs compared with controls (Figure 4C). Collectively, these data suggest that loss of *Mapt* leads to defects in cell organization in vivo.

Previous studies revealed that defects in AT2-to-AT1 differentiation exacerbate alveolar fibrosis after bleomycin-induced injury. Therefore, we sought to assess the consequences of loss of TAU on alveolar repair and fibrosis (Figure 4A). Co-immunostaining for GFP with ACTA2 and TAGLN revealed an increase in myofibroblasts in *Mapt* gRNA–administered lungs compared with NTC lungs (Figure 4, D and E). Moreover, quantification revealed a significant increase in ACTA2-expressing regions in areas that have GFP expression, suggesting that defective repair leads to an increase in fibrosis in these lungs compared with controls (Figure 4F). Additionally, immunostaining and quantification for SFN (early PATS) and LGALS3 (late PATS) on sections collected from bleomycin-injured control and *Mapt* gRNA–administered lungs revealed a significant increase in SFN^+^ and decrease in LGALS3^+^ PATS in *Mapt*-depleted cells (Figure 4, G and H). These data suggest an impairment in alveolar epithelial differentiation in *Mapt*-deleted cells. Furthermore, trichome staining revealed an increase in collagen deposition in bleomycin-injured *Mapt-*deleted lungs compared with controls (Figure 4I). Analysis of bleomycin-injured *Mapt-KO* mice confirmed these findings (Supplemental Figure 6, D–H). Together, these data demonstrate that TAU regulates MT dynamics during AT2 differentiation that is required to ensure AT1 regeneration after injury.

### Loss of TP53 disrupts TAU expression and MT and AT1 organization during AT2-to-AT1 differentiation.

**Figure 5:**
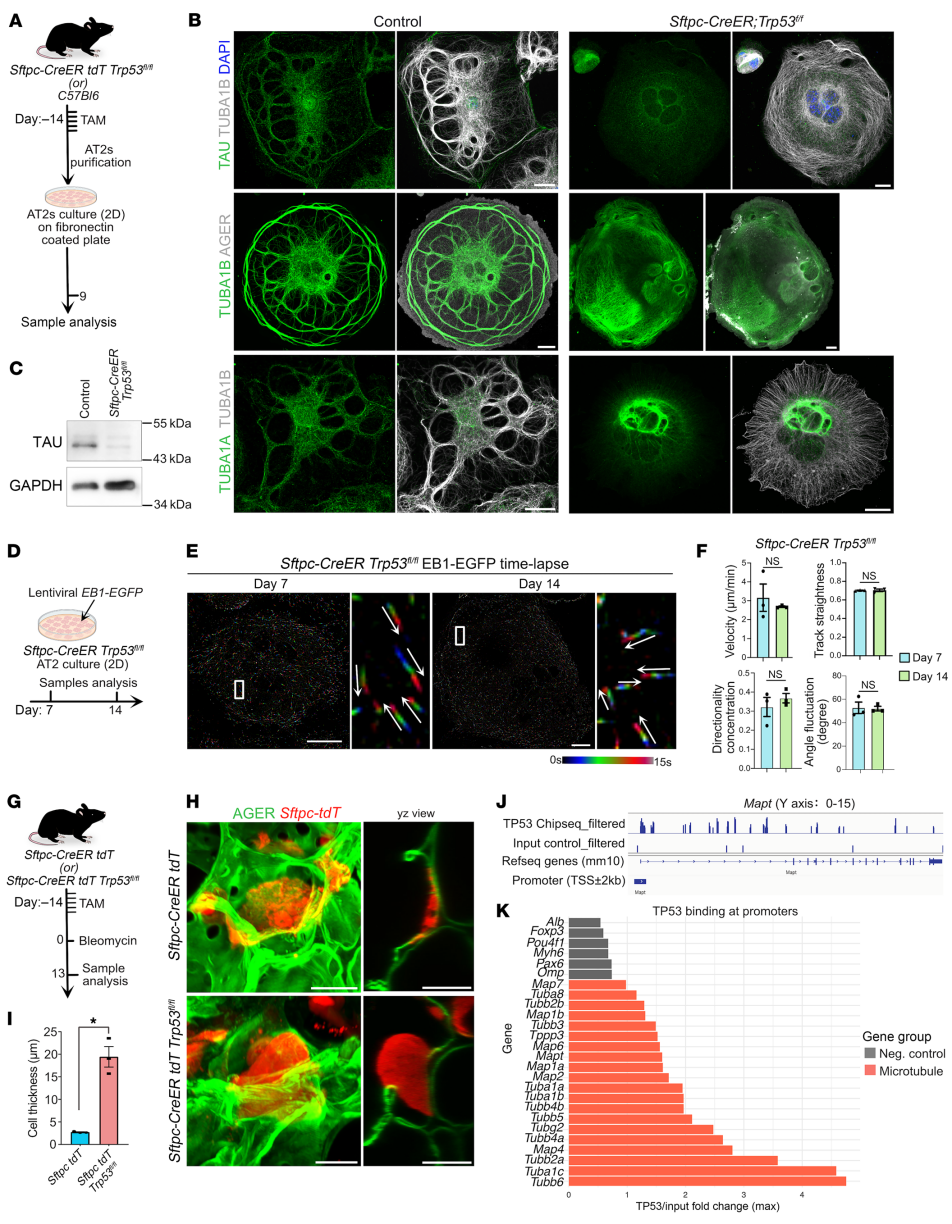
Loss of TP53 disrupts MT organization and AT1 differentiation. (**A**) Experimental workflow for tamoxifen administration to delete TP53 in AT2s followed by AT2 isolation for ex vivo analysis in *Sftpc-CreER R26R-tdTomato Trp53^fl/fl^* or control mice. (**B**) Images showing TAU and tubulin localization in control and TP53-deleted cells. Scale bars: 20 μm. DAPI stains nuclei (blue). (**C**) Western blot of TAU and GAPDH (loading control) in control and TP53-deleted cells. (**D**) Experimental design for *EB1-EGFP* lentivirus administration in TP53-deleted AT2s followed by live imaging on days 7 and 14. (**E**) Kymograph and time-lapse images for EB1-EGFP in *Trp53*-deleted cells on days 7 and 14 of culture. Scale bars: 20 μm. White box indicates region of enlarged image. White arrows indicate direction of growing plus ends of MTs. (**F**) Quantification of EB1-EGFP comet velocity (μm/min), directionality concentration, angle fluctuation (degree), and track straightness in TP53-deleted cells cultured for 7 and 14 days. Unpaired 2-tailed *t* test. (**G**) Experimental workflow for tamoxifen administration to delete TP53 in AT2s followed by bleomycin injury in *Sftpc-tdT-Trp53-KO* or control mice (*Sftpc-tdT*). (**H**) Staining for AGER (green) and tdTomato (red) in bleomycin-injured controls and *Trp53-KO* mice. Scale bars: 20 μm. (**I**) Quantification of cell thickness of lineage-labeled cells in controls and *Trp53-KO* mice following bleomycin injury. *P = 0.017, unpaired 2-tailed *t* test. (**J**) Integrative Genomics Viewer (IGV) tracks show enrichment for TP53 binding in genomic loci corresponding to *Mapt* promoter. (**K**) Graph depicting enrichment of TP53 binding on MT-associated genes (red) and unrelated negative controls (gray). Data in **F** and **I** are presented as mean ± SEM. *n* = 3 biological replicates.

In neurons from Alzheimer’s disease and in certain carcinomas, TP53 and TAU directly interact to control cellular processes, such as DNA damage repair and cellular stress pathways (56). Previous studies have also implicated a role for TP53 in AT2-to-AT1 differentiation after injury (17, 29, 30). To assess the role of TP53 in regulation of TAU and MT assembly, we purified AT2s from *Sftpc-creER R26-tdT Trp53^fl/fl^* (hereafter referred to as *Trp53-KO*) mice that had received tamoxifen. AT2s from C57BL/6 mice served as controls (Figure 5A). Using our 2D cultures, we assessed the ability of AT2s to differentiate into AT1s, as well as MT organization and TAU expression. Immunostaining and Western blot analysis revealed that TAU expression was decreased in *Trp53-KO* cells compared with controls (Figure 5, B and C). Moreover, the localization pattern of TUBA1B and TUBA1A correlated with disorganization of MTs, including the loss of thick MT bundles in *Trp53-KO* cells (Figure 5B). Additionally, immunostaining revealed a decrease in expression of AGER in mutant cells compared with controls (Figure 5B). Of note, mutant cells exhibited more than 2 nuclei, a finding consistent with previous reports that suggested a role for TP53 in regulating γ-tubulin and blocking cytokinesis (57, 58). Consistent with MT disorganization, mutant cells showed an increase in cell thickness and a slight decrease in cell area compared with controls (Figure 5B). To assess MT dynamics, we transduced a lentivirus carrying EB1-EGFP fusion protein into AT2s lacking TP53 (Figure 5D). Time-lapse imaging and comet tracking analyses and velocity, directionality, and angle fluctuation quantification revealed that cells at day 7 showed premature bidirectional movement of MTs from center to cortex, which was maintained at day 14, suggesting that they undergo misdirected growth in mutant cells (Figure 1, K and L; Figure 5, E and F; and Supplemental Videos 4 and 5).

To assess the consequences of TP53 deficiency on alveolar epithelial organization, we utilized *Sftpc-tdT-Trp53-KO* mice. Upon tamoxifen administration, there is concomitant expression of tdTomato and loss of *Trp53* specifically in AT2s. *Sftpc-creER R26-tdT* (hereafter referred to as *Sftpc-tdT*) mice served as a control (Figure 5G). To assess the consequences of TP53 loss on alveolar epithelial cell organization, we administered bleomycin to cause lung injury and collected tissues on day 13 postinjury (Figure 5G). Co-immunostaining for AGER and tdTomato on thick tissue slices followed by confocal 3D reconstruction of alveoli revealed large and thin cells co-expressing tdTomato and AGER in control lungs. In contrast, we observed large, balloon-shaped, tdTomato-expressing cells that lacked AGER in TP53-deficient cells, a phenotype similar to that of TAU-mutant cells (Figure 4B and Figure 5H). Quantification confirmed a significant increase in cell thickness in TP53-deficient cells compared with controls (Figure 5I). To assess whether TP53 directly binds on the *Mapt* genomic locus, we reanalyzed previously described ChIP-seq data from purified PATS (29). Integrative Genomics Viewer (IGV) tracks revealed enrichment of TP53 on *Mapt* promoter (Figure 5J). Additionally, we found TP53 binding on multiple tubulin and MT-associated gene loci (Figure 5K and Supplemental Figure 7A). To further test whether the expression of tubulin- and MT-associated genes is altered in TP53-deficient cells, we utilized previously published scRNA-seq data (17). Pseudobulk RNA expression analysis of these data revealed that the expression of *Map1b*, *Map2*, *Map4*, *Map6*, *Map7*, *Tuba1b*, *Tuba1c*, *Tubb4b*, *Tubb5*, and *Tubb6* was decreased in *Trp53-KO* cells (Supplemental Figure 7B). Additionally, to assess whether TP53 similarly controls tubulin- and MAP-encoding genes in human cells, we reanalyzed publicly available scRNA-seq data from lung adenocarcinoma (59). Although these datasets lack TP53 mutation annotation, the majority of tumor cells exhibit decreased *TP53* transcript levels (consistent with loss-of-function or nonsense mutations). We found that *MAP2*, *MAP4*, *MAP7*, *TUBA1A*, *TUBB4B*, *TUBB6*, and *TUBG2* were downregulated in TP53-low cells (Supplemental Figure 7C). Together, these data point to a mechanism whereby TP53 directly binds and controls tubulin- and MAP-encoding genes during AT2-to-AT1 differentiation.

### TAU expression, localization, and requirement during human AT2 differentiation.

**Figure 6:**
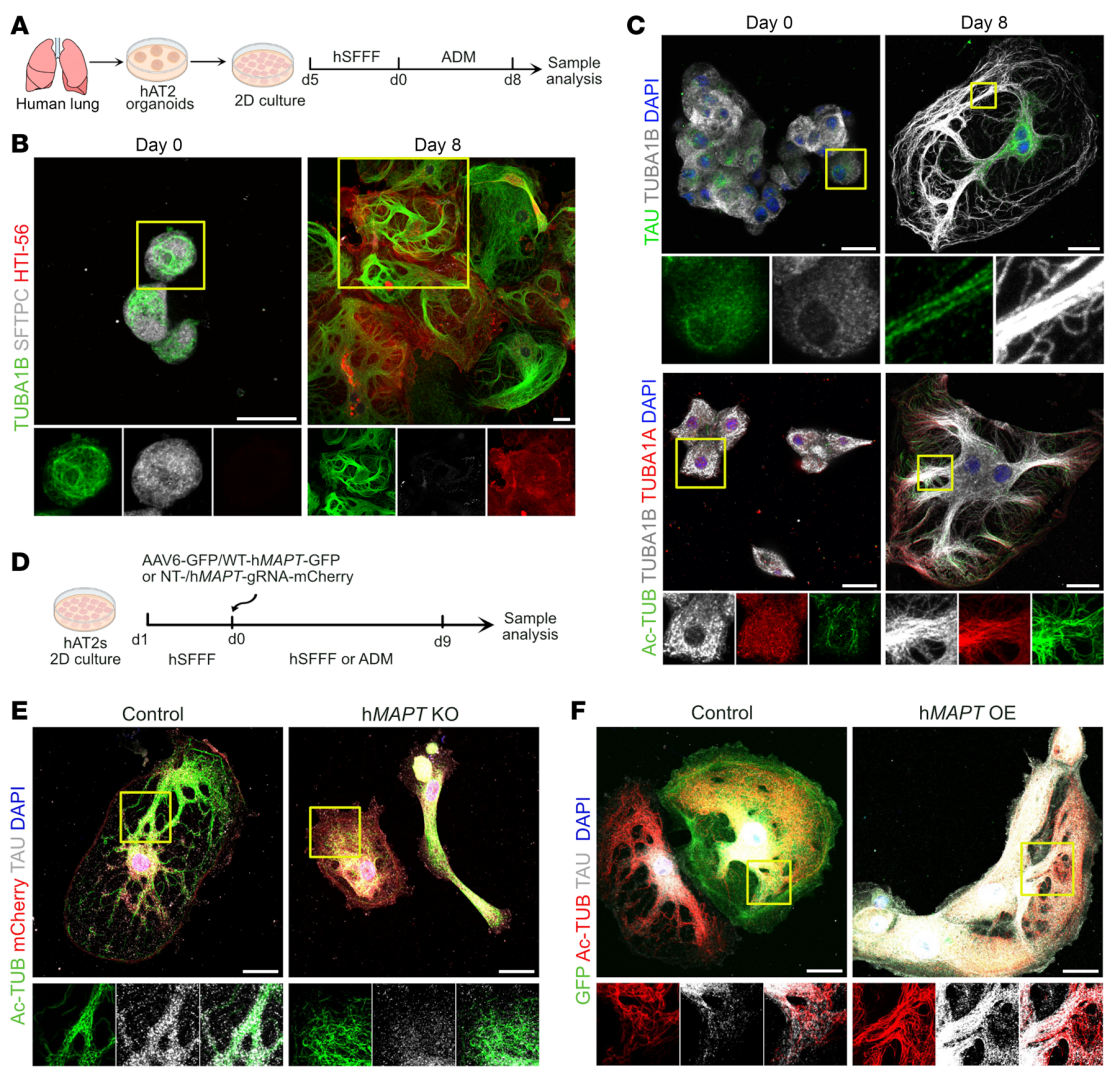
*MAPT* regulates human AT2 differentiation into AT1s. (**A**) Schematic of human AT2 purification, culture, and differentiation followed by analyses. (**B**) Staining for TUBA1B (green), SFTPC (gray), and HTI-56 (red) in AT2s and ex vivo–differentiated AT1s. Scale bars: 20 μm. (**C**) Staining for TAU (green) and TUBA1B (gray) (upper panel) and Ac-TUB (green), TUBA1A (red), and TUBA1B (gray) (lower panel) in AT2s and AT1s. Scale bars: 20 μm. (**D**) Workflow for *MAPT* deletion or overexpression in AT2s followed by differentiation to AT1s and analyses. (**E**) Staining for Ac-TUB (green), mCherry (red), and TAU (gray) on *MAPT*-deleted and control cells. Scale bars: 20 μm. DAPI stains nuclei (blue). (**F**) Staining for GFP (green), Ac-TUB (red), and TAU (gray) on *MAPT*-OE and control cells. Scale bars: 20 μm. Yellow box in merged image indicates region of single-channel images.

We then sought to assess TAU expression, localization, and requirement during human AT2 differentiation. First, we purified human AT2s as previously described and cultured them in serum-free, feeder-free (SFFF) conditions for expansion or in alveolar differentiation medium (ADM) for differentiation into AT1s on plates coated with either collagen or fibronectin as described above (Figure 1A; Supplemental Figure 1, A–C; and Figure 6A). As expected, these culture conditions supported either selective expansion of AT2s or their differentiation into large, thin, and flat AT1s ex vivo as assessed by co-immunostaining for SFTPC and HTI-56, respectively (Figure 6B). Furthermore, immunostaining for TUBA1B revealed the presence of thick MT bundles in AT1s. We then assessed the expression and localization dynamics of TAU at early and late stages in culture. Co-immunostaining for TAU, TUBA1B, TUBA1A, and Ac-TUB revealed a gradual increase in TAU expression as the AT2s differentiate to AT1s. Further, TAU localization changed from random puncta to an organized fiber-like pattern that aligned along the thick MT bundles similar to results seen in mice (Figure 6C).

Second, to test the requirement of TAU for proper differentiation of AT2s into AT1s, we screened and selected a gRNA that can efficiently target human *MAPT* gene (Supplemental Figure 8A). As illustrated in Figure 6D, we generated lentiviral particles expressing Cas9, *MAPT* gRNA, and a fluorescent reporter, mCherry, and transduced them into human AT2s. NTC gRNA served as a control. Transduced cells were then induced to differentiate into AT1s and collected on day 9 postinfection for analysis. Co-immunostaining for mCherry, TAU, and Ac-TUB revealed loss of TAU and absence of thick MT bundles in *MAPT* gRNA–transduced cells compared with NTC gRNA (Figure 6E). Further, we found disorganization of morphology from thin, large, and flat in the case of NTC gRNA–transduced cells to thick and elongated in *MAPT* gRNA–transduced cells. To assess the consequences of TAU gain of function, we transduced AAV6-expressing human *MAPT* and GFP into AT2s. Of note, ectopic expression of TAU in AT2s was not sufficient to induce AT2-to-AT1 differentiation (Supplemental Figure 8B). However, induction of differentiation by administering ADM resulted in the disorganization of cell morphology specifically in ectopic TAU–expressed cells compared with controls as revealed by co-immunostaining for GFP, TAU, and Ac-TUB (Figure 6F). Additionally, ectopic TAU-expressing cells showed abnormal thick MT bundles. Together, both gain and loss of TAU disrupted MT organization and gave rise to thick cells during human AT2-to-AT1 differentiation, similar to what had been observed with mouse cells.

### Genetically regulated MAPT expression within the 17q21.31 haplotype influences pulmonary disease risk.

Common genetic variation at the *MAPT*-containing 17q21.31 locus has been strongly associated with idiopathic pulmonary fibrosis (IPF), chronic obstructive pulmonary disease (COPD), and lung function traits (60–64). More specifically, this locus includes a 900 kb inversion, which contains genetic variation in strong linkage disequilibrium, resulting in the H1 and H2 inversion-tagging haplotypes (65). Consequently, these pulmonary disease associations reflect haplotype-level association, rather than a single SNP. Within the disease-associated haplotype, we found no *MAPT*-nonsynonymous coding variants. Rather, most haplotype variants localized to the *MAPT* locus were noncoding, consistent with the idea that if disease risk is conferred by this locus, it is through *MAPT* expression regulation. To explore this, we examined *MAPT* expression quantitative trait locus (eQTL) data from nasal airway epithelial brushings generated on a childhood asthma cohort (GALA = 681). *MAPT* was identified as a significant nasal eGene, with genetic variation tagging the inversion haplotype associated with *MAPT* expression (Supplemental Figure 8C). Examining *MAPT* expression by one of the eQTL variants, rs1981997, we found that the minor allele (A) was associated with lower *MAPT* expression (Supplemental Figure 8D). Notably, the A allele of rs1981997 has been associated with decreased IPF risk (60). In contrast, based on data reported by the Genotype-Tissue Expression (GTEx) Consortium in lung tissue, the A allele for rs1981997 is associated with increased *MAPT* expression. GTEx also reports rs1981997 as a *MAPT* eQTL across 18 additional tissues, with the direction of effect sometimes matching that of lung tissue and other times matching the nasal pattern. Together, these results support a model whereby genetically regulated *MAPT* expression within the 17q21.31 haplotype influences pulmonary disease risk, with the direction of effect depending on the tissue context.

## Discussion

Efficient diffusion of gases across the alveolar epithelium into the blood capillaries and vice versa requires that these tissues maintain appropriate cell numbers and organization (6, 66). Here, we describe a unique MT organization, in which differentiating AT1s develop thick MT bundles that control cell thickness and area. We speculate that such thick MT bundle organization promotes the expansion of the cytoplasm and decrease in cell thickness that enhances gas diffusion as compared with the radial and dispersed pattern observed in AT2s and other cell types. Furthermore, thick MT bundles likely provide structural support and stability for the thin and expansive AT1s during cyclic breathing movements. Our work uncovered an unexpected role for TAU in alveolar epithelial differentiation. Specifically, TAU seems to undergo condensation and is organized into a beads-on-a-string-like pattern in PATS and in AT1s. We also find that TAU localization precedes thick MT bundle formation, suggesting that TAU initiates MT organization during AT2 differentiation. This aligns with prior studies using in vitro reconstitution assays that revealed TAU droplet formation and localized condensation, which in turn facilitates MT assembly (50). Further, it has been shown that TAU is critical for assembly of well-organized MTs and spacing between bundles in neuronal axons and dendrites (67, 68). Previous studies have implicated that endothelium-derived TAU promotes neuronal tauopathy in *Pseudomonas aeruginosa*–infected mice (69, 70). However, to our knowledge, this is the first report implicating TAU in alveolar epithelial stem cell–mediated repair after injury.

Our data revealed that both gain and loss of TAU disrupted thick MT bundle formation, leading to generation of aberrant differentiated cells with an increase in cell thickness and decrease in cell area. These data suggest that a fine balance in the expression levels of TAU is essential to control thick MT bundle formation and cellular organization. In tauopathies, it has been well documented that hyperphosphorylation and different splice forms of TAU can differently influence MT organization, organelle transport, and mitochondrial function (71). Future studies need to evaluate the role of these different isoforms in alveolar epithelial cells. Interestingly, AT1s share some similarities with oligodendrocytes. For instance, oligodendrocytes generate elaborate myelin sheaths that wrap around neuronal axons, facilitating rapid signal conduction. Both cell types express *Mapt* and generate expansive membrane, which in turn is regulated by MTs and MAPs (72–76). Additionally, both AT1s and oligodendrocytes express the transcription factor MYRF (myelin regulatory factor). Based on this, we propose that both AT1s and oligodendrocytes use similar programs to generate expansive membranes via TAU and organized MT structures.

Our study also revealed that expression of TAU is decreased upon loss of transcription factor TP53 during AT2 differentiation. Aside from its well-known functions in genome stability, DNA damage repair, and cell death pathways, previous studies have also implicated a role for TP53 in regulating cytoskeleton in alveolar epithelial cells (29). We now infer a role for TP53 in regulating TAU expression and thereby MT organization during differentiation of alveolar epithelial cells. This is in line with previous studies that revealed a role for TP53 in directly regulating the expression of MAPs in neurons and other cells (56). Indeed, we find that loss of TP53 leads to altered MT bundle formation and generation of aberrant alveolar epithelial cells with an increase in cell thickness, a phenotype similar to TAU loss of function. These data suggest that TP53/TAU axis controls thick MT bundle formation to control cellular alveolar epithelial cell organization.

Recent GWAS have identified potential risk variants in the *MAPT* locus in COPD and pulmonary fibrosis (60, 61). In addition to *MAPT*, this 17q21 locus harbors other genes, including *KANSL1*, which has been identified as a risk allele in eQTL studies that utilized scRNA-seq and GWAS data to compute risk allele association (77). Our analyses further provide support that variants in *MAPT* locus are associated with IPF disease risk. Together, these data indicate the need to further investigate the TAU association in IPF and COPD.

## Methods

### Sex as a biological variable.

Our study examined male and female animals, and similar findings are reported for both sexes.

### Mouse strains, bleomycin injury, and viral delivery.

Both male and female mice aged 8–16 weeks were used for experiments. All the mice were C57BL/6 unless otherwise indicated. The following mice were used for experiments: WT from The Jackson Laboratory (strain 000664), *Sftpc^tm1(cre/ERT2)Blh^* (*Sftpc-CreER*) (78), *B6.Cg-Gt(ROSA)26Sor^tm14(CAG-tdTomato)Hze^/J* (*R26R-tdTomat*o) (79), *H11-Cas9* (48), *Rtkn2-CreER R26R-Kaleidoscope* (41), *B6.129X1-Mapttm1Hnd/J* (54), and *Trp53^fl/fl^* (80) (mixed background). For lineage tracing mice received 3–5 doses of 2 mg tamoxifen (Sigma-Aldrich) per 20 g of body weight via intraperitoneal injection. For bleomycin-induced lung injury, 2.5 U/kg bleomycin was administered intranasally 2 weeks after tamoxifen injection and the mice were monitored daily. Mice that were administered PBS served as controls. The mice were sacrificed at different times after bleomycin injury. For intranasal AAV infection, mice were anesthetized with 3% isoflurane in an induction chamber followed by 2.5 × 10^10^ viral particles administration resuspended in 60 μL of physiological saline (Henry Schein, 002477).

### Mouse lung tissue dissociation and AT2 cell isolation.

Lung dissociation was performed as described previously (34, 81). Briefly, lungs were inflated with an enzymatic dissociation solution: 450 U/mL Collagenase I (Worthington, LS004197), 5 U/mL Dispase (Corning, 354235), and 0.33U/mL DNase I (Roche, 10104159001). Lung lobes were minced and incubated in enzyme solution at 37°C 25–35 min. Dissociation was quenched with 10% FBS/DMEM and strained. Cell pellet was resuspended in red blood cell lysis buffer (100 μM EDTA, 10 mM KHCO_3_, 155 mM NH_4_Cl) for 2 min, followed by quenching with 10% FBS/DMEM and filtration. For FACS, the cell pellet was resuspended in a sorting buffer (0.5% BSA [Genclone, 25-529F], 2 mM EDTA). Cells were stained with EpCAM/CD326-Brilliant-Violet-711 (BioLegend, 118233, 1:200), Lysotracker-Green DND-26 (Invitrogen, L7526, 1:10,000), CD140a-PE (BioLegend, 135905, 1:200), CD31-eFluor-450 (Invitrogen, 48-0311-82, 1:200), and CD45-eFluor-450 (Invitrogen, 48-0451-82, 1:200). EpCAM^+^Lysotracker^hi^ cells were collected in 2% FBS/DMEM/F12. Sorting was performed using a SONY SH800S or MA900.

### Collagen I, fibronectin, and 5% Matrigel coating.

To maintain AT2s collagen was used. Briefly, 100 μL of Cellmatrix Type I-A (Wako Chemicals, 637-00653) was mixed with 100 μL of DMEM-F12/Ham media, and 20 μL of reconstitution buffer (2.2 g NaHCO_3_ in 100 mL of 0.05N NaOH and 200 mM HEPES) was added. Ice-cold collagen solution was added to wells, spread, and polymerized at 37°C for 30 min. AT2s were plated on collagen-coated wells. To induce mouse AT1 differentiation, AT2s were seeded on fibronectin. First, fibronectin (MilliporeSigma, F4759) was diluted with PBS to a concentration of 50 μg/mL, then added to wells at 37°C for 30 min to 6 hours. Fibronectin was removed, and wells were washed once with PBS followed by mouse AT2 seeding diluted in culture medium. For 2D cultures on Matrigel, AT2s were plated on wells collated with 5% Matrigel (Corning, 354230). Briefly, Matrigel was serially diluted in DMEM/F12 to a concentration of 5%, followed by well-coating at 37°C for 30 min. Next, Matrigel was removed and AT2s were seeded. AT2s were cultured in SFFF medium. The medium was changed every 2 days.

### Mouse AT2 cell expansion.

Mouse AT2 organoids were cultured in SFFF conditions as described previously (34, 81). Briefly, 3,000–5,000 FACS-sorted AT2s were resuspended in SFFF media and mixed with Matrigel in droplet format. After Matrigel solidification at 37°C for 15–20 min, the mouse SFFF medium was added. AT2 organoids were passaged to single cells using TrypLE select (Gibco, 12563029) every 10–12 days.

### Human lung dissociation and AT2 purification.

Human lung dissociation was performed as described previously (34, 81). Briefly, 2–3 g of tissue was washed with PBS/1% Antibiotic-Antimycotic followed by pleura, small airway, and vasculature removal. Remaining tissue was cut into small pieces followed by digestion (Collagenase I: 1.68 mg/mL, Dispase: 5 U/mL, DNase: 10 U/mL) at 37°C for 1–1.5 hours. Cells were filtered and rinsed with 10% FBS/DMEM. Cell suspension was spun down at 450*g* for 10 min, and the pellet was resuspended in red blood cell lysis buffer (Thermo Fisher Scientific, A1049201) for 5 min, washed with 10% FBS/DMEM, filtered, and pelleted. Approximately 2 million–10 million cells were resuspended in MACS buffer (PBS, 1% BSA, 2 mM EDTA) as per manufacturer’s instructions and incubated with TruStain-FcX (BioLegend, 422032) for 15 min at 4ºC followed by mouse HTII-280 (1:60 dilution) antibody for 1 hour at 4ºC. Cells were washed twice with MACS buffer and incubated with anti-mouse IgM microbeads for 15 min at 4ºC, loaded into the LS-column (Miltenyi Biotec, 130-042-401), and collected magnetically.

### Human AT2 cell culture and cell differentiation.

Human AT2 cultures were performed as previously described (34, 81). Human AT2 organoids were cultured in SFFF conditions in 50% Matrigel. For differentiation, AT2s were dissociated and plated in 5% Matrigel and cultured in SFFF media for 3–5 days followed by 7–8 days of ADM media replacement containing 10% human serum.

### EB1-EGFP lentivirus transduction.

Lentivirus production was performed as described previously with modification (82). Briefly, 70%–80% confluent HEK293T cells from ATCC (CRL-1573) were prepared in 10% FBS/DMEM/1% penicillin-streptomycin. Two hours before transfection, the medium was changed to 5% FBS/DMEM without penicillin-streptomycin followed by transfection with 10 μg of pLenti-EB1-EGFP (Addgene, plasmid 118084), 7 μg psPAX2 (Addgene, plasmid 12260), and 5 μg pCMV-VSV-G (Addgene, plasmid 8454) plasmids using PEI Max (1:4) (Polysciences, 24765). After overnight incubation, the medium was changed to 10% FBS/DMEM/1% penicillin-streptomycin. Viral supernatant was collected 48, 72, and 96 hours after transfection followed by virus concentration using Lenti-X Concentrator (Takara, 631231). The viral pellet was dissolved in DMEM/F12 and titrated using a qPCR lentivirus titter kit (Applied Biological Materials, LV900). Single-cell suspensions of mouse AT2s were resuspended in SFFF containing lentivirus at 1:100 and seeded on a fibronectin-coated, glass-bottom dish (Matsunami Glass, D35-14-1-U). Cells were incubated with lentivirus overnight followed by SFFF replacement.

### Live cell imaging of EB1-EGFP signal in mouse AT2s and kymograph analysis.

Virus-infected mouse AT2s were recorded on days 7 and 14 at 1.5-second intervals. For kymograph analysis, the time series stack data were applied to the Fiji plugin software: *tubeness* to remove background signals followed by *KymoResliceWide* analysis according to the distributor’s guide. Analysis including velocity, directionality concentration, angle fluctuation, and track straightness were performed. Briefly, images were converted to 8-bit. A region of interest was manually defined within the cell boundary. To enhance linear comet signals, the *tubeness* filter was applied. Tracking of EB1 comets was performed using the TrackMate plugin (Simple LAP tracker). All quantitative analyses were performed in R (packages: tidyverse, readr, ggplot2, circular). For each EB1 comet track, the *XY* displacement and duration were used to calculate velocity (μm/min) and movement angle (degrees). Directionality concentration (DC) was calculated using circular statistics to quantify the uniformity of comet movement angles, with higher DC values indicating more coherent orientation. Angle fluctuation was calculated as the standard deviation of frame-to-frame directional changes, reflecting local instability. Track straightness, defined as the ratio of net displacement to total path length, was quantified to evaluate the linearity of EB1 comet trajectories. All measurements were calibrated using the imaging scale (μm/pixel) and frame interval (s/frame).

### Vector cloning of AAV-CRISPR–KO plasmids.

Candidate gRNA sequences were picked up from Brie library (46) or designed using CHOPCHOP (83). Two oligos containing sgRNA sequences [Oligo 1: ACC+5′gRNA(20-mer)3′, Oligo 2: AAC+5′Reverse complement of gRNA(20-mer)3′] were obtained and annealed using T4PNK (New England Biolabs [NEB] M0201S) according to the manufacturing protocol. Backbone plasmid pAAV-U6-sgRNA-CMV-GFP (Addgene, 85451) was cut with restriction enzyme Sap1 (NEB, R0569S), and a larger size of cut plasmid was extracted from the gel. Finally, the annealed oligo was ligated to backbone plasmid using Quick ligase (NEB, M2200S).

### AAV production and transduction.

AAV production and transduction were performed as previously described (55). Briefly, 70%–80% confluent HEK293T cells were prepared in 10% FBS/DMEM/1% penicillin-streptomycin. Two hours before transfection, medium was changed to 5% FBS/DMEM without penicillin-streptomycin, and cells were transfected using PEI Max (1:4) with 50 μg of transgene plasmid, 100 μg of adenovirus helper plasmid (XX680), and 50 μg of AAV serotype plasmid. Following overnight incubation, the medium was replaced to 5% FBS/DMEM/1% penicillin-streptomycin. Viral supernatant was collected 4 days after transfection and purified by iodixanol gradient using Opti-prep Density Gradient Medium (Sigma, D1556) and ultracentrifuge (60,000 rpm). Titers of virus were measured by qPCR with primers amplifying the AAV2 ITR regions (fw:5′-AACATGCTACGCAGAGAGGGAGTGG-3′; rev:5′-CATGAGACAAGGAACCCCTAGTGATGGAG-3′). For AAV transgene transduction to ex vivo culture, AAV supernatant was diluted with SFFF medium at a ratio of 1:4 to 1:5 without concentration and administered to cells.

### RNA preparation and bulk RNA-seq.

For total RNA extraction, cells were resuspended in TRIzol (Thermo Fisher Scientific, 15596026), and total RNA was extracted using Direct-zol RNA Microprep Kit (Zymo, R2061) according to the manufacturer’s protocol. Bulk RNA-seq was conducted on samples with RIN values greater than 8.0 using a Bioanalyzer (Agilent). Ribosomal RNA from total RNA samples (100 μg) was depleted using NEBNext rRNA Depletion Kitv2 (NEB, E7400L). Libraries were prepared using NEBNext Ultra II Directional RNA Library Prep Kit for Illumina (NEB, 7760S).

### Reanalysis of scRNA-seq data.

Line plots of relative gene expression were performed by reanalyzing the available data (NCBI GEO GSE141259) (30). We extracted gene expression trajectory data from the converging trajectories using the interactive web tool (https://theislab.github.io/LungInjuryRegeneration/).

### Bulk RNA-seq and differential gene expression analysis.

Purified RNA (1 μg) from each sample was enriched for Poly-A RNA using NEBNext Poly(A) mRNA Magnetic Isolation Module (NEB, E7490). Libraries were prepared using NEBNext Ultra II RNA Library Prep Kit for Illumina (NEB, E7770). Paired-end sequencing (150 bp for each read) was performed using HiSeq X (Illumina) with at least 15 million reads per sample. Quality of sequenced reads was assessed using FastQC (https://www.bioinformatics.babraham.ac.uk/projects/fastqc/). PolyA/T tails were trimmed using Cutadapt (84). Adaptor sequences were trimmed and reads shorter than 24 bp were trimmed using Trimmomatic (85). Normalization and extraction of differentially expressed genes between samples were performed using an R package, DESeq2 (86).

### ChIP-seq signal filtering and visualization.

To visualize TP53 binding enrichment across MT-related genes, published ChIP-seq data for TP53 (NCBI GEO GSE141635; CTGF^+^tdTomato^+^ PATS) and corresponding Input control were processed using R (v4.3.2). Signal tracks in bedGraph format were imported via the rtracklayer package and converted into BigWig files after filtering by signal intensity and genomic coordinates for each gene were obtained from TxDb.Mmusculus.UCSC.mm10.knownGene and org.Mm.eg.db. ChIP and Input signals overlapping each gene region were extracted using subset ByOverlaps. Peaks with signal intensity greater than 5.29 were selected corresponding to the 95th percentile of the Input signal distribution. Only peaks exceeding this percentile were considered TP53 enriched relative to the Input control. The resulting BigWig files were loaded into IGV (v2.17.0) to visualize TP53 ChIP enrichment relative to Input across the analyzed MT-related genes.

### Reanalysis of human and mouse scRNA-seq datasets to assess TP53-dependent regulation of AT1-associated MT gene programs.

Reanalysis of publicly available scRNA-seq datasets — human lung adenocarcinoma (GSE131907) (59) and a Kras-driven mouse lung cancer model (GSE231681) (17) — was conducted in Seurat v5.0.1. Briefly, for the human dataset, raw UMI matrices and cell annotations were filtered. Data were normalized, highly variable genes were selected, and PCA was performed. Cell type annotations from the original study were incorporated as metadata, and AT1s were extracted. Malignant epithelial cells were stratified based on TP53 expression, and those with TP53 expression below the median were defined as TP53-low malignant. For the mouse dataset, raw HDF5 matrices were imported and filtered. Samples representing KT (*Trp53* WT), KPT (*Trp53* loss), and KFT (*Trp53* hyperactive) were merged and normalized, and the top 2,000 variable genes were identified. Scaled data were subjected to PCA, and principal components 1–30 were used for UMAP embedding and clustering. Cluster identities were assigned using canonical markers. AGER-positive AT1-like cells were extracted for analyses. KFT samples were excluded from KT–KPT comparisons. MAP and tubulin isoform genes were analyzed, and violin plots were generated.

### GWAS data analyses.

*MAPT* eQTL data based on nasal brushings were obtained from a published genome-wide GALA nasal eQTL analysis (87). The *MAPT* LocusZoom plot was constructed using the locuszoomr R package (88), where LD patterns were generated relative to the lead variant using LDlinkR based on the 1000 Genomes Project European population (89). Publicly available eQTL data were examined using the GTEx version 10 portal (https://gtexportal.org/home/).

### Lung tissue fixation and sectioning.

Mouse lungs were inflated and fixed in 4% paraformaldehyde (PFA) at 4°C for 4–6 hours. Lung lobes were separated and washed in PBS followed by incubation in 30% sucrose overnight at 4°C. Lobes were incubated in 1:1 30% sucrose/OCT for 1 hour followed by embedding in OCT blocks and cryosectioning at 8–10 μm thickness.

### Immunostaining on lung sections.

OCT sections were washed with PBS. Antigen retrieval was performed using 10 mM sodium citrate buffer at 90°C–95°C for 15 min. Sections were washed with PBS, permeabilized in PBST (0.1% Triton X-100 in PBS), and incubated with 1% BSA in PBST for 30 min at room temperature (RT) followed by primary antibodies at 4°C overnight. Sections were then washed 3 times (3x) in PBST, incubated with secondary antibodies in blocking buffer for 1 hour at RT, washed with PBST 3x, and mounted using Fluor G with DAPI (Invitrogen, catalog 00-4959-52).

### Immunostaining of cultured cells.

Cultured cells were fixed with 4% PFA at RT for 15 min or with methanol at –20°C for 10 min. Samples were washed with PBS, permeabilized in 0.2% Triton X-100 in PBS, and incubated with 1% BSA in PBS for 30 min at RT, followed by primary antibodies at 4°C overnight. Samples were then washed 3x in PBST, incubated with secondary antibodies for 1 hour at RT, washed with PBST 3x, and mounted.

### Precision cut lung slices (PCLS) and immunostaining of PCLS.

Mouse lungs were inflated with 2% low-melting agarose dissolved in PBS as previously described (90). PCLS (300 μm) were obtained using compresstome (PRECISIONARY, VF510-Z). For immunostaining, PCLS were fixed in 4% PFA at 4°C for 1 hour. Sections were washed with PBS, permeabilized in 0.3% Triton X-100 in PBS, and incubated with blocking buffer (1% BSA, 0.3% Triton X-100 in PBS) for 1 hour at RT followed by primary antibodies at 4°C overnight. Sections were then washed 3x in wash buffer (0.5% Tween-20, 0.5% Triton X-100 in PBS), incubated with secondary antibodies in blocking buffer at 4°C overnight, and washed 3x in wash buffer and twice in PBS before imaging on a glass-bottom dish. Three-dimensional rendering of acquired stack images was performed using Imaris (Oxford Instruments) or Icy software (https://icy.bioimageanalysis.org).

### Protein extraction and Western blot analysis.

Cultured cells were washed with ice-cold PBS and collected in cell lysis buffer (50 mM Tris-HCl at pH 7.5, 150 mM NaCl, 1% Triton X-100, 2 mM EDTA, and 2 mM DTT and protease inhibitor cocktail). Following a 15-minute incubation on ice, the lysates were spun down at 13,000*g* for 15 min, and the supernatant was collected for a Bradford analysis (Bio-Rad Laboratories). Samples were prepared in Laemmli buffer, boiled for 10 min at 95°C, and loaded on 12% SDS-PAGE gels followed by transfer, blocking in 5% milk for 1 hour at RT, incubation with primary antibodies overnight at 4°C, washes with TBST, and incubation with secondary antibodies. The following primary and secondary antibodies were used: anti-Tau (10274-1-AP, Proteintech, 1:1,000), anti-GAPDH (GT239, GeneTex, 1:10,000), anti-Rabbit IgG-HRP (4030-05, Southern Biotech, 1:10,000), anti-Mouse IgG-HRP (1030-05, Southern Biotech, 1:10,000), Alexa Fluor 594 donkey anti-goat (A11058, Invitrogen), Alexa Fluor 488 donkey anti-goat (A11055, Invitrogen), Alexa Fluor 488 donkey anti-rabbit (A21206, Invitrogen), Alexa Fluor 647 donkey anti-rabbit IgG (A31573, Invitrogen), Alexa Fluor 488 donkey anti-rat (A21208, Invitrogen), Alexa Fluor 594 donkey anti-rat (Invitrogen, A2109), Alexa Fluor 488 donkey anti-mouse (Invitrogen, A21202), and Alexa Fluor 594 donkey anti-mouse (Invitrogen, A21203). Signals were detected using a Pierce ECL-2 (Thermo Fisher Scientific). Band intensities were quantified using ImageJ (NIH).

### Imaging of mitochondria and tubulin in mouse AT1s.

Cultured AT1s were incubated for 30 min at 37°C in SFFF media containing Tubulin Tracker Green (T34075, Invitrogen, 1:4,000) and PKmito Orange Dye (CY-SC053, Cytoskeleton-Inc., 1:5,000), followed by a 5-minute wash in SFFF containing 1 μg/mL Hoechst-33342 stain. Cells were rinsed 3x in SFFF media and imaged.

### Masson’s trichrome staining.

Trichrome staining was performed using a Masson Trichrome Staining Kit (HT15-1KT, Sigma-Aldrich) and a Weigert’s Iron Hematoxylin Set (HT1079-1SET, Sigma-Aldrich) according to manufacturer protocols on OCT-frozen sections (Thermo Fisher Scientific). Images were recorded using a 20× objective of the Axio imager (ZEISS).

### Antibodies.

The following antibodies and dyes were applied to samples for immunostaining: anti-RAGE/AGER (MAB1179, R&D Systems, 1:500), anti-proSP-C (AB3786, MilliporeSigma, 1:500), anti-ABCA3 (3C9) (sc58220, Santa Cruz Biotechnology, 1:300), anti-Claudin4 (36-4800, Invitrogen, 1:200), anti-Actin, alpha-Smooth Muscle Cy3-conjugated (C6198, MilliporeSigma,1:500), anti-HT1-56 (TB-29AHT1-56, Terrace Biotech, 1:300), anti-HTII-280 (TB-27AHT2-280, Terrace Biotech, 1:50), anti-GFP (NB100-1770, Novus Biologicals, 1:500), anti-tdTomato (AB8181-200, OriGene, 1:1,000), anti-TUBA1A antibody (PA5-22060, Invitrogen, 1:100), anti-TUBA1B (66031-1-Ig, Proteintech, 1:500), anti-acetylated Tubulin (66200-1-Ig, Proteintech, 1:500), anti-Tau (10274-1-AP, Proteintech, 1:200), anti-Tau-1 (PC1C6) (MAB3420, MilliporeSigma,1:100), anti-Tau (Tau-5) (AHB0042, Invitrogen,1:50), anti-MAP2 (17490-1-AP, Proteintech, 1:500), anti-Keratin8 (TROMA-I, DSHB, 1:50), anti-GM130 (610822, BD Biosciences, 1:50), Alexa Fluor-555 Phalloidin (A34055, Invitrogen, 1:400), Alexa Fluor-647 Phalloidin (A22287, Invitrogen, 1:400), LEL-DyLight-649 (DL-1178, Vector Laboratories,1:1,500), anti-FLAG-M2 (F1804, Sigma-Aldrich, 1;1,000), anti-Calnexin (AB22595, Abcam, 1:500), anti-CD107a/LAMP-1 (121601, BioLegend, 1:500), anti-SFN (PA5-95056, Invitrogen, 1:250), anti-LGALS3-Alexa647 (125408, BioLegend, 1:500), and anti-TAGLN/Transgelin (ab14106, Abcam, 1:250).

### Image acquisition, processing, and quantification.

Images were captured using an Olympus FV3000 confocal microscope with 20×, 30×, 40×, and 60× objectives. For long-term live imaging Olympus VivaView FL Incubator Microscope was used with a 20× objective. Images were processed using the Olympus CellSens application or ImageJ, and figures were prepared using Affinity Designer. Measurements and quantifications were performed using ImageJ-Fiji using a sample of biological replicates (*n* = 3).

### Statistics.

Statistical methods relevant to each figure are outlined in each figure legend. Sample size was not predetermined. Data are presented as means with SEM. Statistical analysis was performed in Microsoft Excel, GraphPad Prism, and R. A 2-tailed Student’s *t* test was used for the comparison between 2 experimental conditions. We used Shapiro-Wilk analyses to test whether data were normally distributed and used Mann-Whitney *U* test for the comparison between 2 conditions that showed non-normal distributions. One-way ANOVA was used for comparison between more than 2 conditions.

### Study approval.

The animal experiments were approved by the Duke University Institutional Animal Care and Use Committee in accordance with the US NIH *Guide for the Care and Use of Laboratory Animals* (National Academies Press, 2011). Healthy human lungs were obtained in accordance with Institutional Review Board oversight: Duke University Pro00114526 — exempt research as described in 45 CFR 46.102(f), 21 CFR 56.102(e) and 21 CFR 812.3(p), which satisfies the Privacy Rule as described in 45CFR164.514.

### Data availability.

All quantification values represented in the graphs are provided in the Supporting Data Values file. Further information and resources are available upon request. Bulk RNA-seq data of cultured cells have been deposited at NCBI GEO (GSE287523) and are publicly available as of the date of publication.

## Author contributions

SK and KE were designated co–first authors, as SK led the initial study design, experiments, and manuscript drafting, whereas KE led the major revision, including key experimental design, execution, and analysis. SL performed experiments and analyzed data. NM, YK, and PA performed transcriptome data analysis. AS assisted in immunostaining experiments. VH and JC provided lungs from *Rtkn2-CreER R26R-Kaleidoscope* mice. MAS, JW, and NDJ performed GWAS analyses. AT co-designed and supervised the work, performed image acquisition, co-wrote the manuscript, and prepared figures. PRT conceived, co-designed, and supervised the work and co-wrote the manuscript. All authors reviewed and edited the manuscript.

## Supplementary Material

Supplemental data

Unedited blot and gel images

Supplemental video 1

Supplemental video 2

Supplemental video 3

Supplemental video 4

Supplemental video 5

Supporting data values

## Version 1 02/05/2026

In-Press Preview

## Version 2 04/01/2026

Electronic publication
