# Supplementary material for: TP53/TAU axis regulates microtubule bundling to control alveolar stem cell–mediated regeneration
Source: J Clin Invest. 2026 Feb 5;136(7):e194762. doi: 10.1172/JCI194762 (PMC13038196; doi:10.1172/JCI194762)
Supplement: Supplemental data [file jci-136-194762-s191.pdf]

Supplemental Figures

Supplemental Figure 1

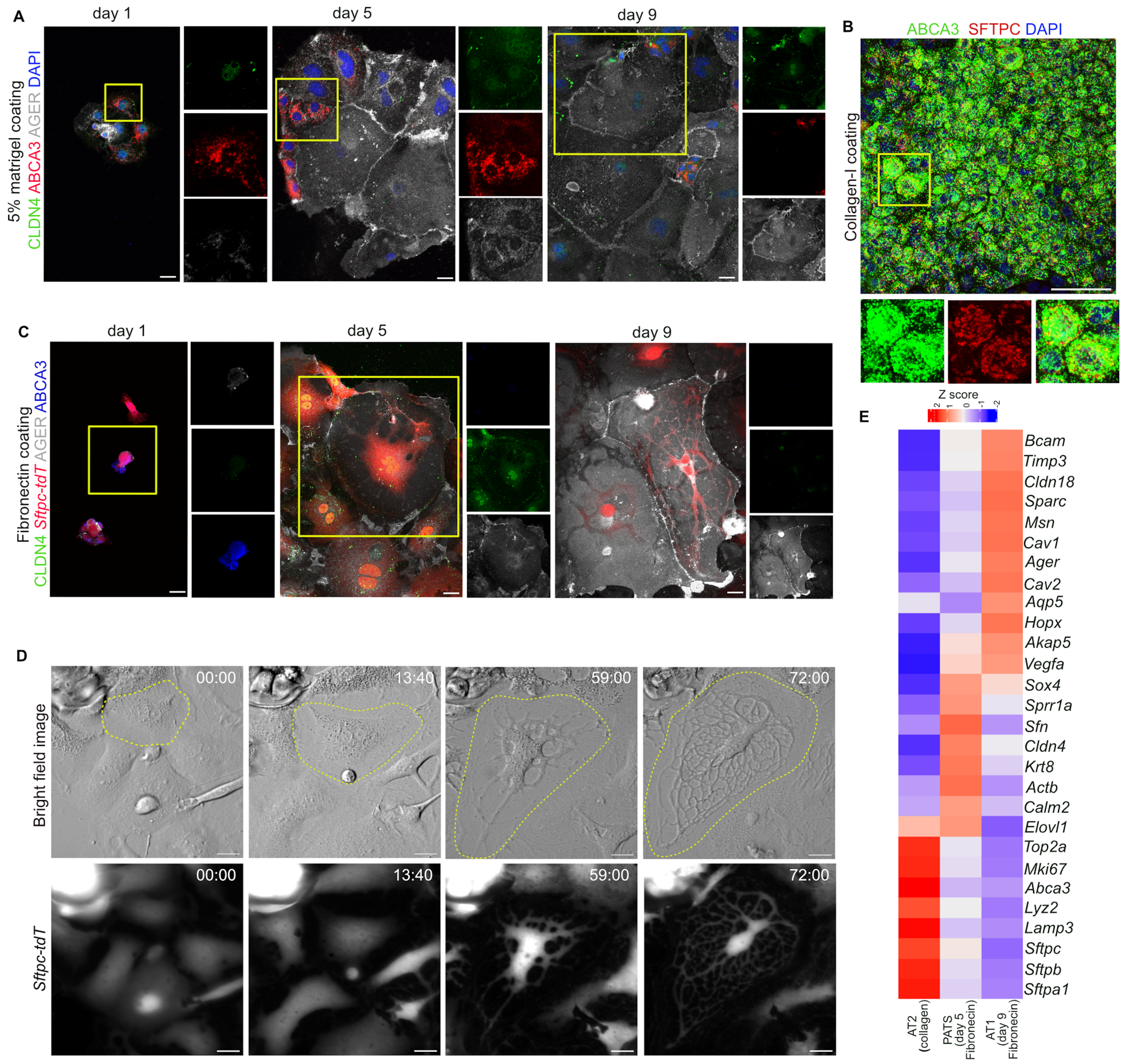

Supplemental Figure 1. The establishment and characterization of *ex vivo* 2D alveolar epithelial cells culture conditions. (A) Immunostaining for CLDN4 (green), ABCA3 (red) and AGER (grey) on alveolar epithelial cells cultured on 5% Matrigel for different days. Scale bars: 20μm. (B) Staining for AT2 markers, ABCA3 (green) and SFTPC (red), in AT2s cultured on Collagen I for 5 days. Scale bar: 50μm. (C) Staining for CLDN4 (green), ABCA3 (blue), and AGER (grey) on AT2s isolated from *Sftpc-CreER;R26R-tdTomato* (red) lungs and cultured on fibronectin for different days. Scale bars: 20μm. (A and B) DAPI stains nuclei (blue). Yellow box indicates region of single channel images. (D) Time frames showing the expansion of cultured alveolar

epithelial cells isolated from over time. Scale bars: 10μm. Yellow dashed line depict expanding cell. (E) Heatmap showing expression of AT2, PATS and AT1 markers in cells collected from indicated culture conditions.

Supplemental Figure 2

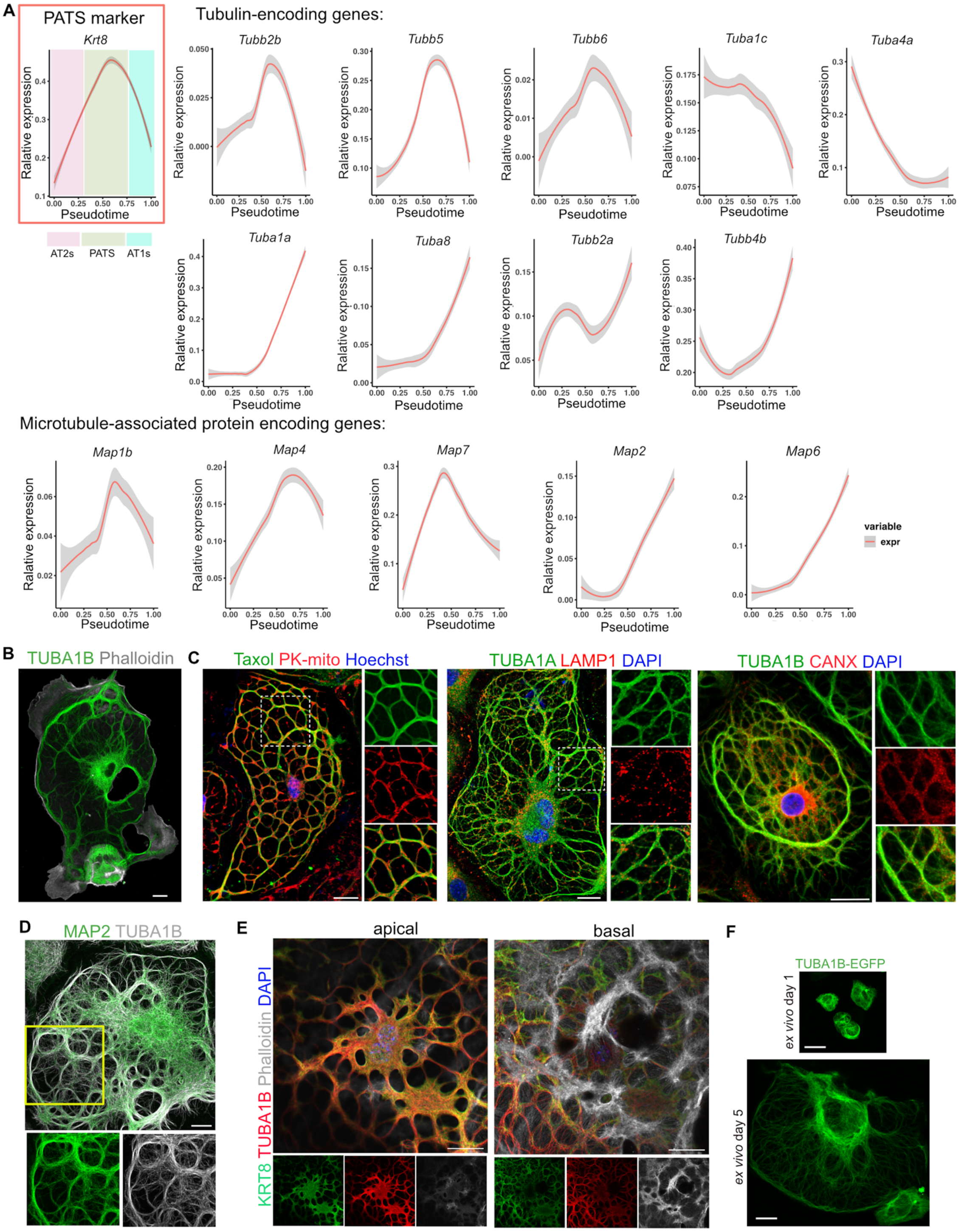

Supplemental Figure 2. Dynamics expression of tubulin-encoding genes and microtubule-associated protein encoding genes during AT2 differentiation to AT1. (A) Pseudotime analysis visualizing expression dynamics of tubulin-encoding genes and microtubule-associated protein encoding genes during AT2-AT1 differentiation. *Krt8* expression is depicting emergence of PATS. The pseudotime for AT2, PATS and AT1s were labelled manually based on expression of *Krt8.* (B) Staining for TUBA1B (green) and phalloidin (grey) in the day 9 cultured cells. Scale bar: 20µm. (C) Staining for Taxol-tubulin (green), PK-mito (red, mitochondria), TUBA1A (green), LAMP1 (red, lysosomes), and CANX (red, endoplasmic reticulum). Hoechst and DAPI stain nuclei (blue). Scale bar: 20µm. (D) Staining for MAP2 (green) and TUBA1B (grey) at day-9 of culture. Scale bar: 20 µm. (E) Images showing localization of KRT8 (green), TUBA1B (red) and phalloidin (grey) on apical and basal side of the AT1s. Scale bar: 20µm. (F) Representative images showing TUBA1B-EGFP in cultured AT2 (culture day-1) and PATS (culture day-5). Scale bars: 20µm.

Supplemental Figure 3

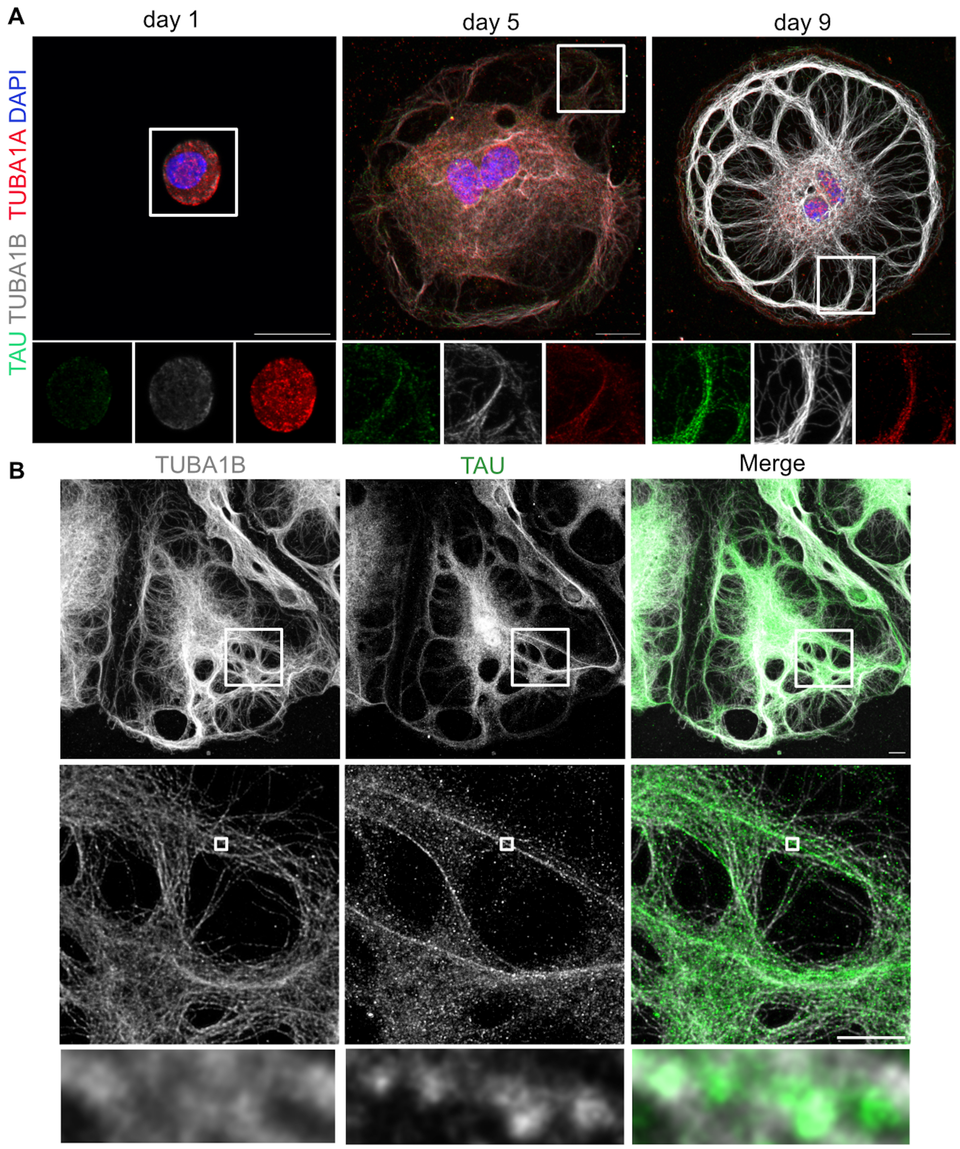

Supplemental Figure 3. Localization of TAU in AT2s and AT1s. (A) Immunostaining for TAU (green), TUBA1B (grey) and TUBA1A (red) during AT2-to-AT1 differentiation. Scale bar: 20μm. (B) Staining for TUBA1B (grey) and TAU (green) in AT1s. Scale bars: 20μm. White box in merged image indicates region of single channel images.

Supplemental Figure 4

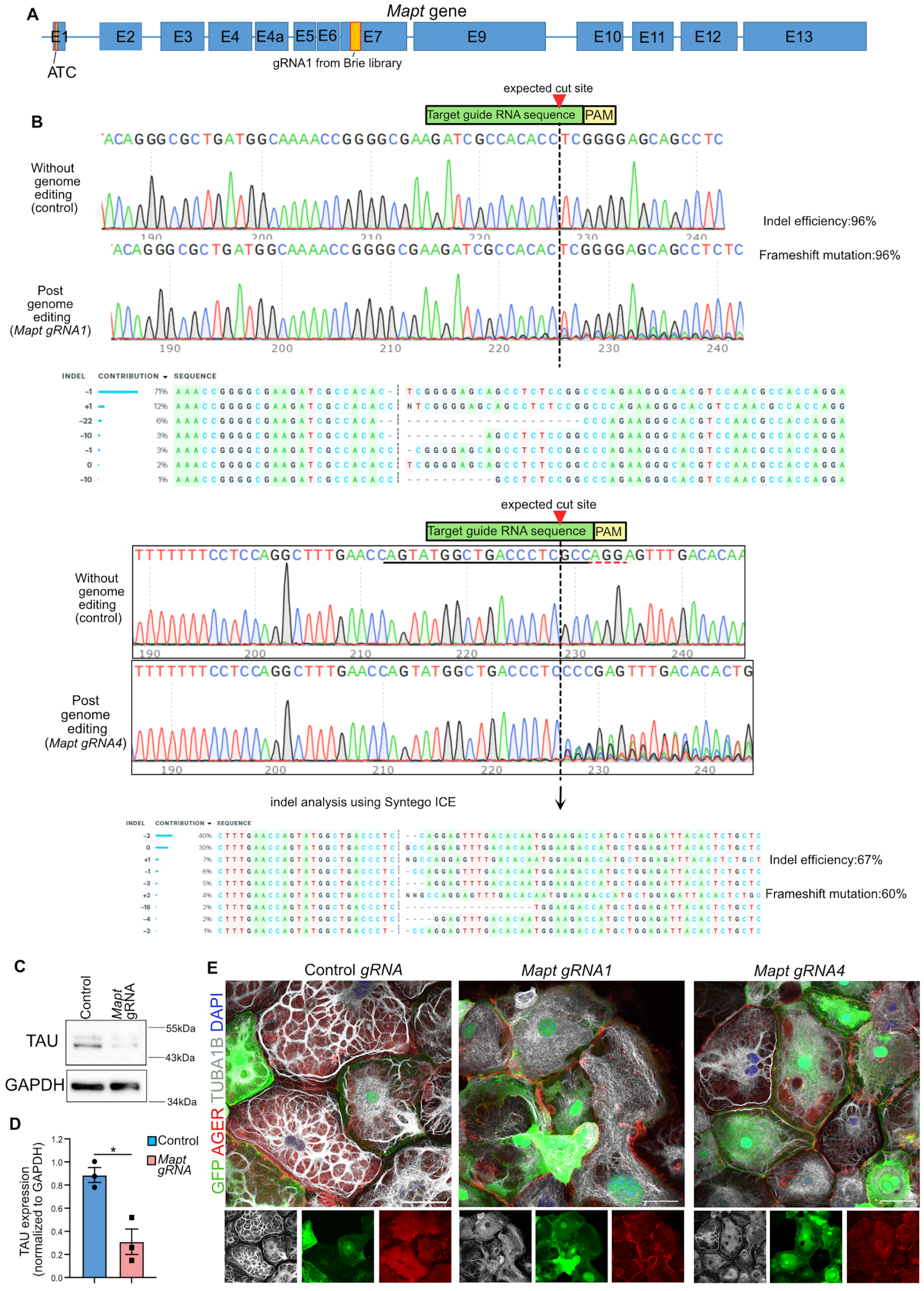

Supplemental Figure 4. Characterization of mouse *Mapt* gRNA efficiency. (A) Schematic of *Mapt* gene. (B) Representative Sanger chromatograms and indel efficiency analysis in controls and *Mapt gRNA1* and *Mapt gRNA4* edited cells. (C) Western blot of TAU in control and *Mapt*-deleted cells. GAPDH was used as a loading control. (D) Quantification of TAU expression in control and *Mapt*-deleted cells. *$p<0.05$, unpaired t-test. n=3 biological replicates. (E) Staining for GFP (green, gRNA delivered AT2s) and AGER (red) and TUBA1B (grey) in control and *Mapt*-deleted AT2s. Scale bars: 50μm.

Supplemental Figure 5

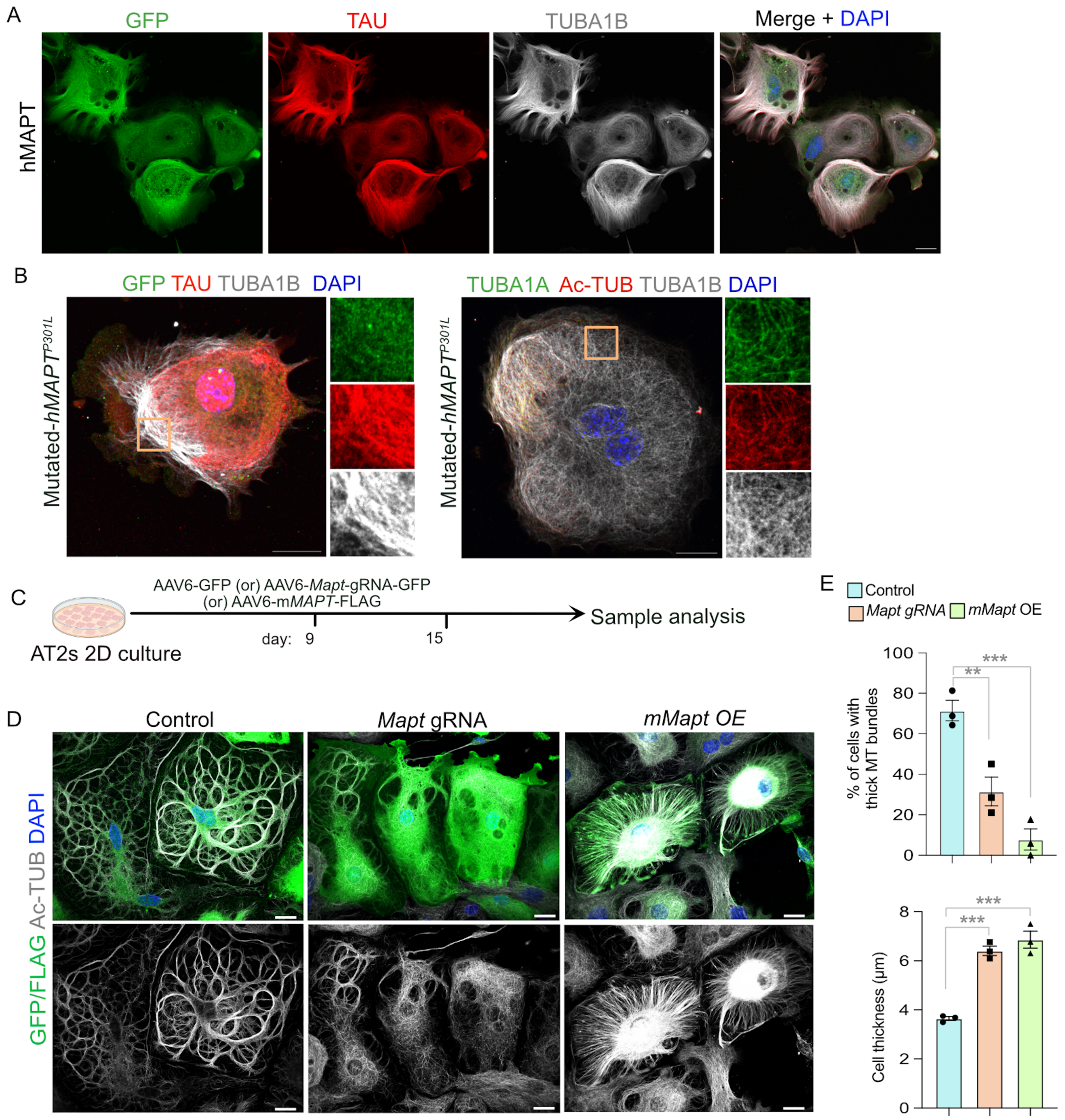

Supplemental Figure 5. Ectopic expression of WT hMAPT or mutated hMAPT^{P301L} and *Mapt-KO* disrupts thick MT-bundle formation. (A) Staining for GFP (green), TAU (red) and TUBA1B (grey) in hMAPT-overexpressed cell (green). Scale bars: 20µm. (B) Staining for GFP (green), TAU (red) and TUBA1B (grey) (left image) and TUBA1A (green), Ac-TUB (red) and TUBA1B (grey) in hMAPT^{P301L} overexpressed cells. DAPI stains nuclei (blue). (C) Experimental workflow for AT2-AT1 differentiation followed by *AAV6-Mapt* gRNA or *AAV6-Mapt-OE* administration and sample collection. (D) Staining for Ac-TUB (grey) in virus infected (green) *Mapt-KO*, *Mapt*-OE, and control cells. (E) Percentage of transduced cells exhibiting thick MT-bundles and quantification of cell thickness. ** p=0.005, *** p<0.001. one-way ANOVA. Data are presented as mean ± s.e.m. n=3 biological replicates.

Supplemental Figure 6

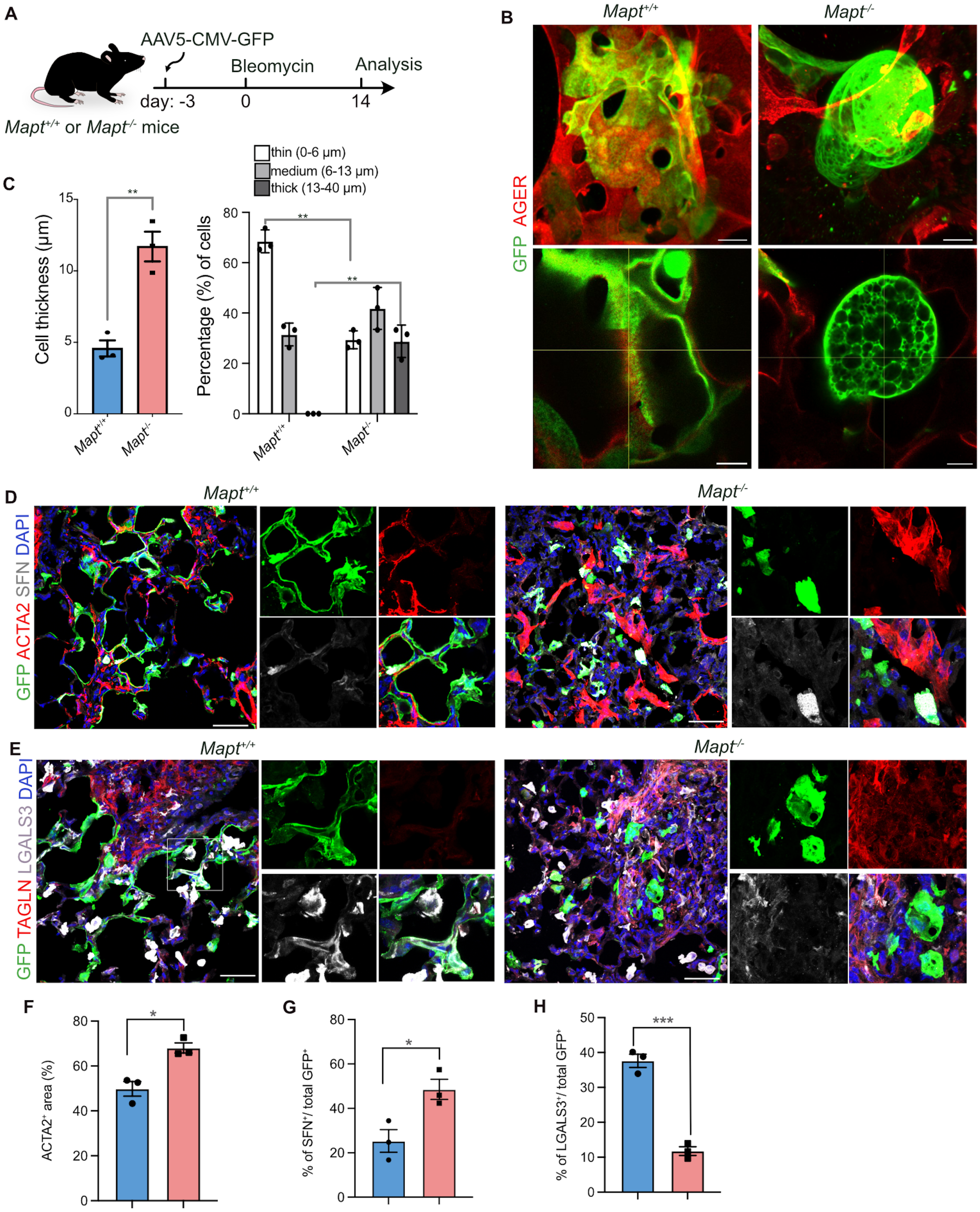

Supplemental Figure 6. *Mapt* deficient AT2s exhibit balloon-like cell morphology in response to bleomycin-induced lung injury. (A) Schematic of experimental workflow for AT2 labelling using AAV5-GFP followed by bleomycin injury for lung sample collection from controls and *Mapt-/-* mice. (B) Staining for GFP (green) and AGER (red) in controls and *Mapt-/-* mice after bleomycin injury. Scale bars: 20μm. (C) Quantification on cell thickness and the distribution of

GFP$^+$ cells with different thickness in wild type control and *Mapt-/-* lungs after bleomycin injury. $^{**}p<0.005$, unpaired two-tailed t-test. (D) Staining for GFP (green), ACTA2 (red) and SFN (grey) in controls and *Mapt-/-* mice after bleomycin injury. Scale bars: 50μm. (E) Staining for GFP (green), TAGLN (red), and LGALS3 (grey) in controls and *Mapt*-deleted AT2s after bleomycin injury. Scale bars: 50μm. DAPI stains nuclei (blue). (F) Quantification of ACTA2$^+$ area of the bleomycin injured lungs. $^*p<0.05$ unpaired t-test. (G) Quantification of SFN$^+$ cells within all GFP$^+$ cells in bleomycin injured lungs. $^*p<0.05$, unpaired t-test. (H) Quantification of LGALS3$^+$ cells within all GFP$^+$ cells in bleomycin injured lungs. $^{***}p<0.001$, unpaired t-test. Data in C, F, G and H are presented as mean ± s.e.m. n=3 biological replicates.

Supplemental Figure 7

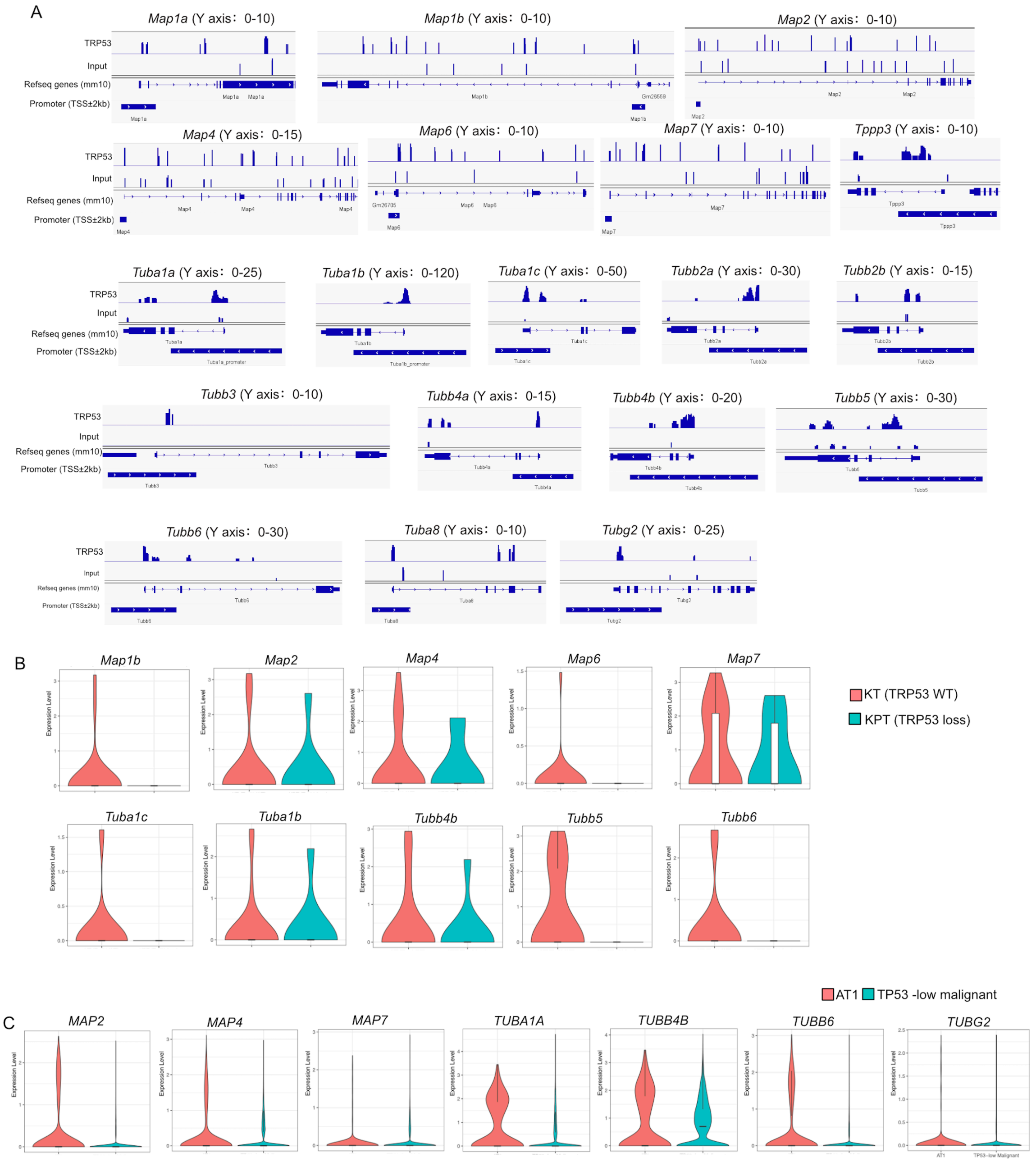

Supplemental Figure 7. **TP53 directly binds on promoters of distinct microtubule related genes and regulates their expression.** (A) IGV tracks show significant enrichment for TRP53 binding in genomic loci corresponding to indicated microtubule associated genes. (B) Violin plots showing the expression of indicated genes in AGER-positive AT1-like cells from KT (TRP53 WT) and KPT (TRP53 loss) lungs in the Kras-driven mouse lung cancer. (C) Violin plots showing the expression of indicated gens in AT1s and TP53-low malignant epithelial cells from the human lung adenocarcinoma. All plots display log-normalized RNA expression values.

Supplemental Figure 8

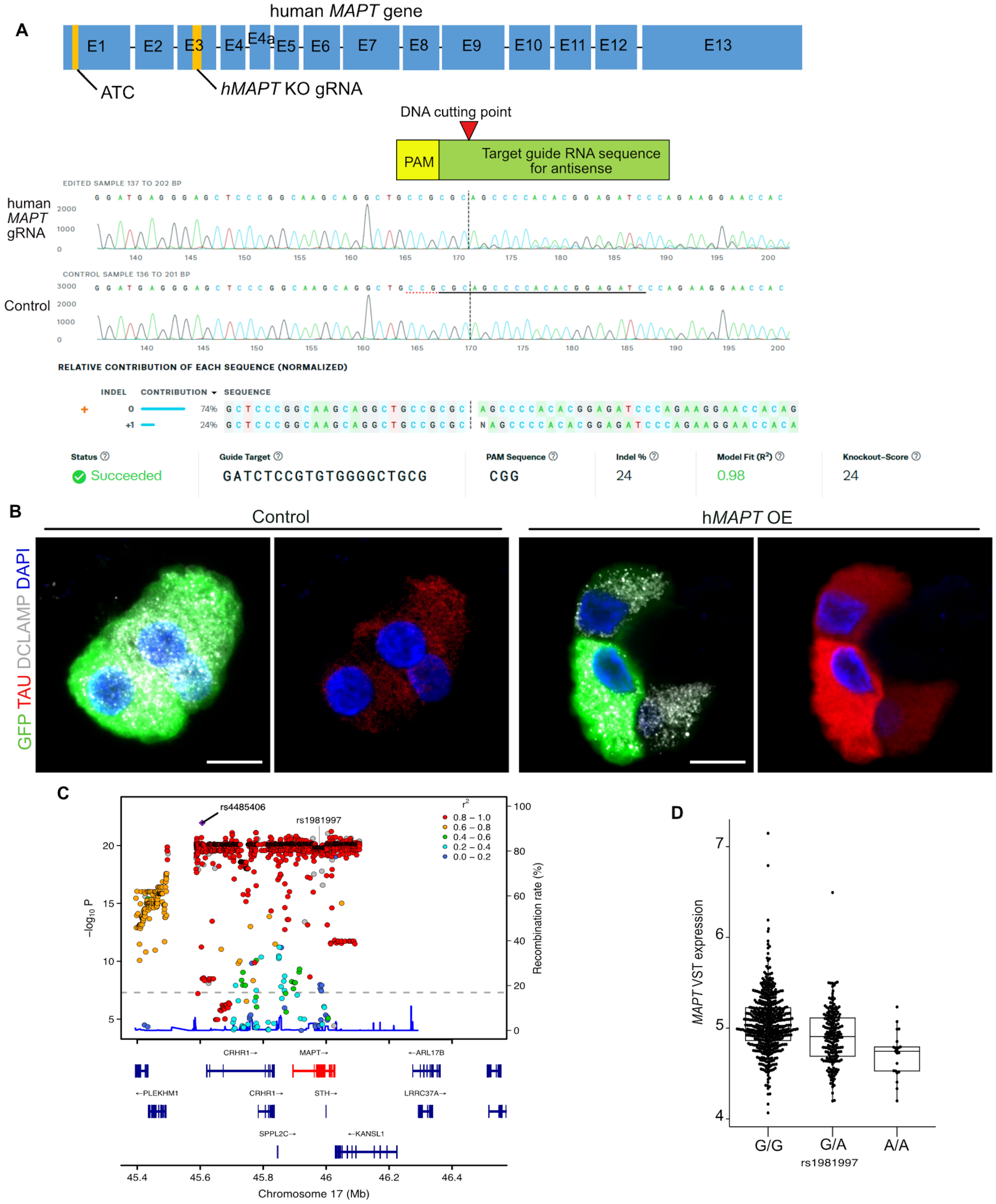

Supplemental Figure 8. Characterization of human *MAPT* gRNA efficiency, MAPT gain of function and GWAS analyses. (A) Representative Sanger chromatograms and indel efficiency analysis in controls and h*Mapt gRNA* edited cells*.* (B) Staining for GFP (green), TAU (red) and an AT2 marker-DC-LAMP (grey), on *MAPT*-overexpressed and control hAT2s. Scale bars: 20µm. DAPI stains nuclei (blue). (C) *MAPT* cis-eQTL Locus Zoom plot of nasal airway brushing data from the GALA cohort, showing a strong LD block marking the *MAPT* eQTL. Linkage disequilibrium is centered on the lead eQTL SNP, rs4485406. (D) *MAPT* expression in nasal brushings stratified by genotype at the IPF risk variant, rs1981997.

Videos Legends

Video 1. Morphological changes in differentiating alveolar epithelial cells. Bright field and epifluorescence (*tdTomato*) showing differentiating alveolar epithelial cells.

Video 2. Tubulin dynamic in transitional epithelial states. Time-lapse images illustrating tubulin dynamics (EB1-EGFP) and orientation in cells cultured for 7-days.

Video 3. Tubulin dynamic in AT1s. Time-lapse images illustrating tubulin dynamics and orientation in cells cultured for 14-days.

Video 4. Tubulin dynamic in *Trp53*-KO alveolar epithelial cells cultured for 7-days. Time-lapse images illustrating tubulin dynamics and orientation in cells cultured for 7-days.

Video 5. Loss of *Trp53* in alveolar epithelial cells leads to disrupted tubulin dynamics in *ex vivo* day-14 cultures. Time-lapse images illustrating tubulin dynamics and orientation in cells cultured for 14-days.
