# Supplementary material for: TP53/TAU axis regulates microtubule bundling to control alveolar stem cell–mediated regeneration
Source: J Clin Invest. 2026 Feb 5;136(7):e194762. doi: 10.1172/JCI194762 (PMC13038196; doi:10.1172/JCI194762)
Supplement: Unedited blot and gel images [file jci-136-194762-s192.pdf]

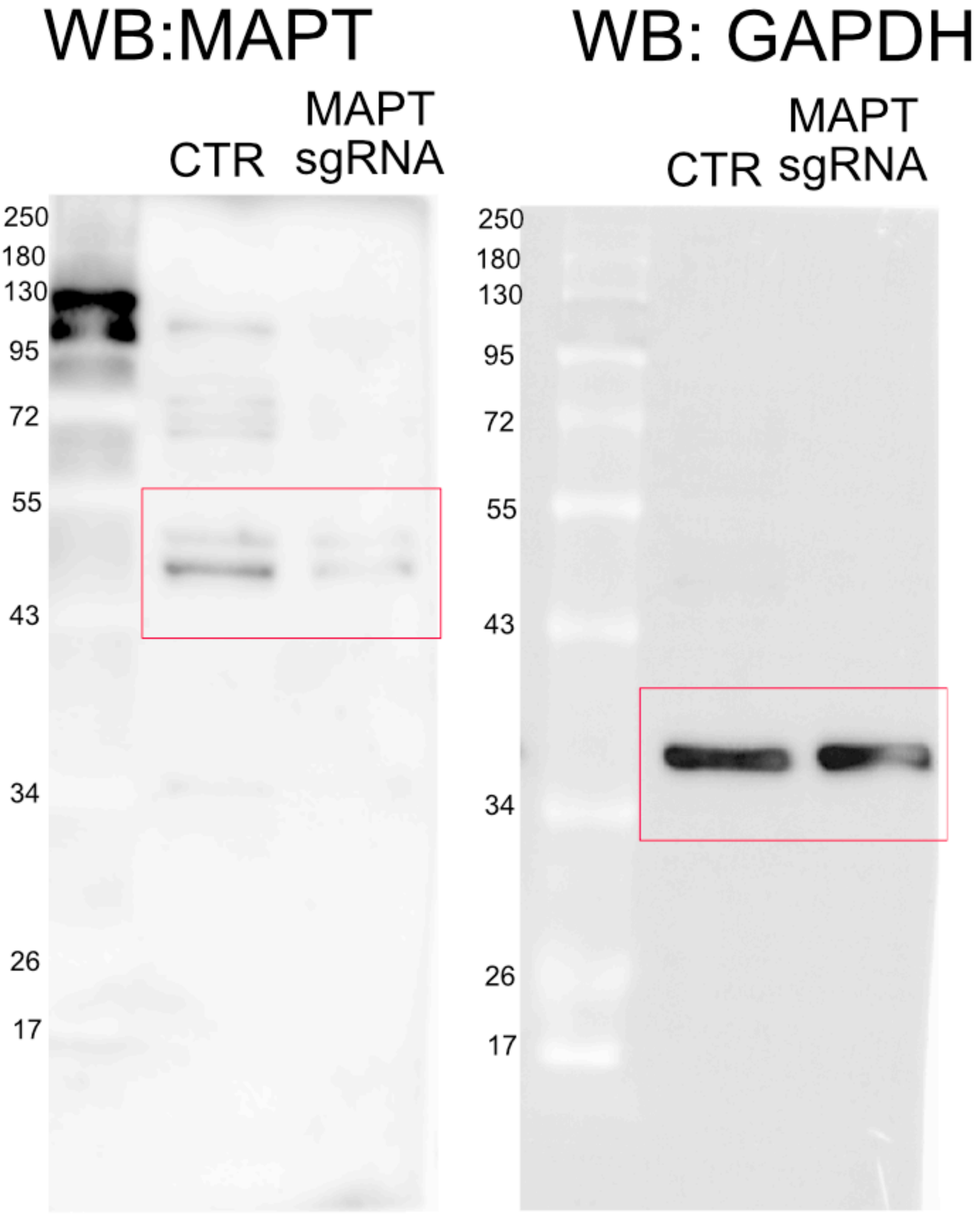
WB:MAPT
CTR
MAPT sgRNA
250
180
130
95
72
55
43
34
26
17
WB: GAPDH
CTR
MAPT sgRNA
250
180
130
95
72
55
43
34
26
17

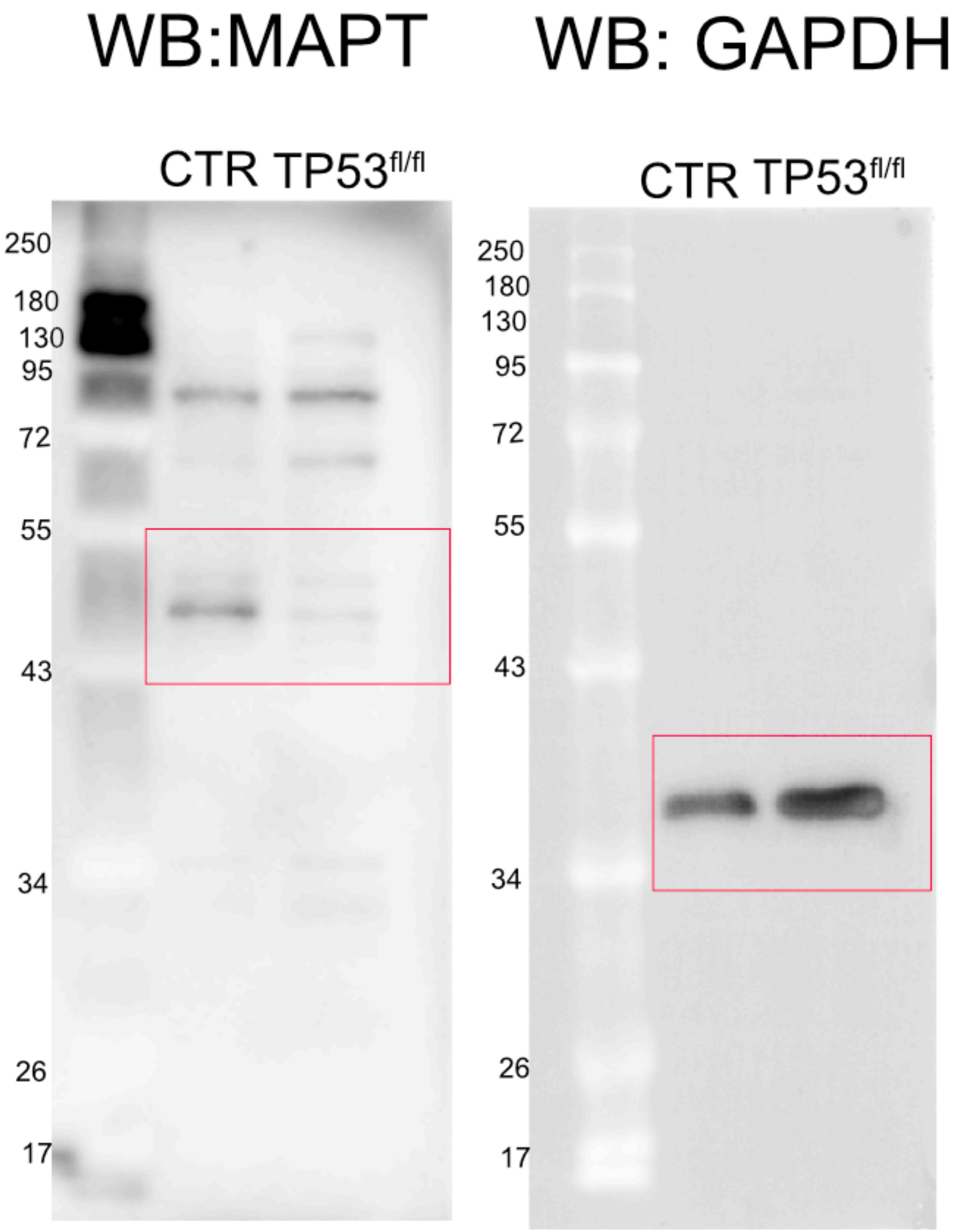
WB:MAPT
CTR TP53fl/fl
250
180
130
95
72
55
43
34
26
17
WB: GAPDH
CTR TP53fl/fl
250
180
130
95
72
55
43
34
26
17
